# Prediction of Gene Activity in Early B Cell Development Based on an Integrative Multi-Omics Analysis

**DOI:** 10.4172/jpb.1000302

**Published:** 2014-02-17

**Authors:** Mohammad Heydarian, Teresa Romeo Luperchio, Jevon Cutler, Christopher J. Mitchell, Min-Sik Kim, Akhilesh Pandey, Barbara Sollner-Webb, Karen Reddy

**Affiliations:** 1 Johns Hopkins University, Department of Biological Chemistry, 725 North Wolfe Street, Baltimore, USA; 2 Johns Hopkins University, Center for Epigenetics, 855 North Wolfe Street, Baltimore, USA; 3 Johns Hopkins University, McKusick-Nathans Institute of Genetic Medicine, 733 North, Broadway Avenue, Baltimore, USA

**Keywords:** Immuno-precipitation, B cell lymphocyte, Post-transcriptional mechanisms, Multi-potent progenitors, RNA-seq analysis

## Abstract

An increasingly common method for predicting gene activity is genome-wide chromatin immuno-precipitation of ‘active’ chromatin modifications followed by massively parallel sequencing (ChIP-seq). In order to understand better the relationship between developmentally regulated chromatin landscapes and regulation of early B cell development, we determined how differentially active promoter regions were able to predict relative RNA and protein levels at the pre-pro-B and pro-B stages. Herein, we describe a novel ChIP-seq quantification method (cRPKM) to identify active promoters and a multi-omics approach that compares promoter chromatin status with ongoing active transcription (GRO-seq), steady state mRNA (RNA-seq), inferred mRNA stability, and relative proteome abundance measurements (iTRAQ). We demonstrate that active chromatin modifications at promoters are good indicators of transcription and steady state mRNA levels. Moreover, we found that promoters with active chromatin modifications exclusively in one of these cell states frequently predicted the differential abundance of proteins. However, we found that many genes whose promoters have non-differential but active chromatin modifications also displayed changes in abundance of their cognate proteins. As expected, this large class of developmentally and differentially regulated proteins that was uncoupled from chromatin status used mostly post-transcriptional mechanisms. Strikingly, the most differentially abundant protein in our B-cell development system, 2410004B18Rik, was regulated by a post-transcriptional mechanism, which further analyses indicated was mediated by a micro-RNA. These data highlight how this integrated multi-omics data set can be a useful resource in uncovering regulatory mechanisms. This data can be accessed at: https://usegalaxy.org/u/thereddylab/p/prediction-of-gene-activity-based-on-an-integrative-multi-omics-analysis

## Introduction

**Figure 1:**
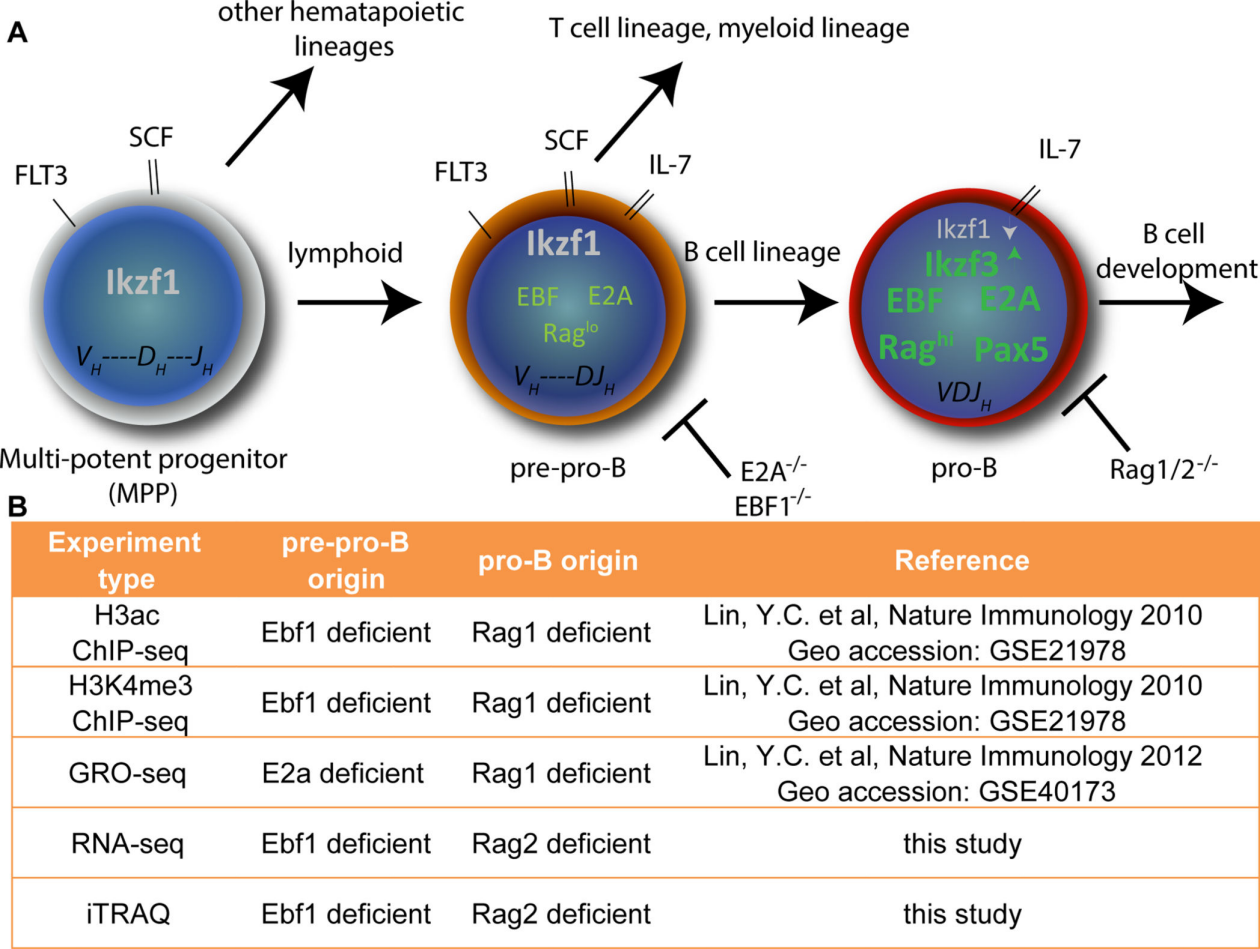
Experimental system and multi-omics data (A) Schematic of early B cell development through three stages: MPP, pre-pro-B, and pro-B cells. Relevant receptors and protein expression are indicated. (B) Multi-’omics’ data used in this study and their respective sources.

Murine B cell development is a leading developmental system for the analysis of gene-regulatory networks that orchestrate cell fate ‘choice’ and lineage commitment [1–3]. Since progenitor cells and discrete developmental intermediates can be isolated and experimentally manipulated, the lymphoid system is particularly well suited for studies involving epigenetic, transcriptional, and protein networks that control cell fate choice. The analyses of early B cell development are thus highly advanced, as regulatory networks of signaling molecules and transcription factors have been elucidated that endeavor to account for the generation of B lineage progeny from multi-potent progenitors (MPP). Importantly, genetically altered mouse lines that harbor deletions in key B cell development factors exist, enabling *ex vivo* expansion of cells arrested at discrete points during lymphopoiesis and B cell specification (Figure 1) [4].

Published work suggests that B lymphocytes develop from lymphoid-primed multi-potent progenitors (LMPPs) in the bone marrow that also give rise to myeloid progeny such as macrophages and granulocytes [3,4]. Restriction of these LMPPs to the B lineage (B cell specification) is controlled by the coordinate activity of a number of transcription factors, including E2a (Tcf3 [transcription factor 3]) and Ebf1 (early B-cell factor 1) which regulate, among other things, rearrangement of the immunoglobulin heavy chain (*Igh*) locus and pre-B cell receptor expression (Figure 1) [5–7]. The *E2a* gene encodes two basic helix-loop-helix isoforms, E12 and E47, generated by alternative splicing [8,9]. Ebf1 is an atypical helix-loop-helix zinc finger protein which, in the hematopoietic system, is expressed exclusively in B lineage cells [10]. Targeted inactivation/deletion of either *E2a* or *Ebf1* leads to a blockade of B cell development at the onset of expression of early B lineage genes, which is the stage at which DNA rearrangements occur between the D to J regions of the *Igh* locus (LMPP or pre-pro-B stage, Figure 1) [11–14]. Cells lacking either E2a or Ebf1 can be cultured *ex vivo* in the presence of Scf (Stem cell factor), Flt3l (Fms-related Tyrosine Kinase 3 Ligand) and Il-7 (Interleukin-7). E2a and Ebf1 both function to activate transcription of several early B lineage genes (Figure 1), and cells lacking these transcription factors are arrested at the pre-pro-B cell stage and do not express key B cell factors, such as Pax-5 (paired-box 5) or Ikzf3 (Aiolos) [4]. In contrast, Rag (Recombination Activating Gene) proteins are necessary for recombination of immunoglobulin genes, and deletion of *Rag1* or *Rag2* leads to a complete block of *Igh* rearrangement and a developmental block at the pro-B cell stage. Ebf1-and E2a–initiated programs to specify B cell developmental progression are intact in these Rag deficient pro-B cells [15,16]. Specifically, Pax-5, Ikzf3 and other key B cell specification factors are present. Importantly, these cells no longer rely on Scf or Flt3l for *ex vivo* survival and the cognate receptors are down-regulated, although they remain dependent on Il-7. Thus, *E2a* or *Ebf1* deletion leads to an early block at the pre-pro-B cell stage, while *Rag* disruption causes a block at the committed pro-B cell stage. These two stages of B lymphopoiesis can be compared to discern key regulatory molecules and events that enable specification to the B cell fate, i.e. the transition from a multi-potent progenitor (pre-pro-B) to a committed B lineage cell (pro-B) (Figure 1).

Using such genetically arrested cells and a ChIP-seq experimental approach, it has recently been determined how Ebf1 and E2a contribute to an altered epigenetic landscape to specify lymphoid cells to the B cell lineage [5]. These results suggest that during the transition from pre-pro-B cell to pro-B cells, enhancers of E2a–regulated genes become mono-methylated on lysine 4 of histone H3 (H3K4me1). Subsequently, the transcription factors Ebf1 and Foxo1 (Forkhead box protein O1) are involved in accumulation of active histone modifications such as tri-methylation on lysine 4 of histone H3 (H3K4me3) at promoters of B cell-specifying genes [5]. In addition, findings from the same laboratory have shed light on Ebf1-mediated epigenetic regulation of its target genes [5]. Ebf1 targets were classified as ‘activated’, ‘repressed’ or ‘primed’ genes and it was observed that, in pro-B and pre-B cells (a later stage in B cell development), the ‘activated’ genes are enriched in H3K4me3 and acetylation on lysine 9 and 14 of histone H3 (H3ac) active chromatin modifications and show low levels on tri-methylated lysine 27 of histone H3 (H3K27me3), a repressive chromatin modification [5]. In contrast, ‘repressed’ genes show the opposite pattern of histone modifications, while enhancers of ‘primed’ genes are enriched for H3K4me1. Thus, the chromatin state of developmentally regulated genes appears to be coincident with their developmental potential. In addition, Global Run-On followed by massively parallel sequencing (GRO-seq) data, which measures ongoing transcription of genes performed on both *E2a deficient* (pre-pro-B) and *Rag1 deficient* (pro-B) cells has demonstrated differential transcription levels at of key developmental genes between these two cells types [17].

Te deep-sequencing technologies enabling elucidation of transcription and chromatin linked developmental programs are indeed a boon in understanding complex developmental programs and much has been gleaned from studying *differential* expression and chromatin status in mediating developmental progression. There is a recurring theme in such epigenetic studies of regulatory elements, however: the supposition that there is a direct link between the changing chromatin state at the promoters of a set of genes and developmental progression. Indeed, to date, almost all studies in developmental systems have focused on chromatin state alone, mRNA levels alone or, at most, a general intersection of chromatin state data with RNA expression data. We hypothesized that assessing the link between changes in chromatin state of protein-coding gene promoters and changes in protein abundance between two distinct stages of B cell development would yield a more refined picture of the link between the changing chromatin state of promoters and the real developmental programs experienced by the cells. We note that other studies have indicated that promoter activity status does not necessarily indicate a change in steady state RNA [18,19], however, we propose to expand on these previous studies by systematically investigating how well correlated *differential* promoter chromatin state is with concordant *differential* ongoing transcription and steady state mRNA levels, as well as *differential* protein levels. By generating and analyzing relative protein abundance and steady state RNA-seq data from pre-pro-B and pro-B cells, along with previously published chromatin and ongoing transcription data in the same cell types, we investigated the link between ongoing transcription, steady state mRNA, inferred mRNA stability and protein levels relative to promoter chromatin state. Our data confirm that active chromatin at gene promoters is a good indicator of appreciable levels of transcription and steady state mRNA and that differentially active chromatin states at promoters are generally good indicators of differential transcription and differential steady state mRNA levels. We further show that chromatin state changes at gene promoters frequently lead to changes in protein abundance. However, many differentially abundant proteins derive from genes whose promoters display active chromatin in both cellular states, generally due to various forms of post-transcriptional regulation. Taken together, these data shed light on differential regulation of proteins that may impact B cell development, but have been missed by ChIP-seq or RNA-based methods alone.

## Methods

### Cell culture

*Ebf1 −/−* pre-pro-B cells and *Rag2 −/−* pro-B cells were cultured on OP-9 stromal cells in Opti-MEM, supplemented with 5% defined FBS (HyClone SH30070.03), 2 mM glutamine, 1% penicillin/streptomycin, beta-mercaptoethanol (1:1000 dilution) and cytokines [pre-pro-B cells: Il-7 (5 ng/mL; Cell Signaling Technology 5217SC), Scf (10 ng/mL; Cell Signaling Technology 5223SC), Flt3l (10 ng/mL; R&D systems 427-FL-025); pro-B cells: Il-7 (5 ng/mL)] under standard tissue culture conditions [3]. Cells were harvested by washing media over stromal cells to liberate B cells, which were then placed in a fresh tissue culture flask to allow residual stromal cells to attach to the culture vessel for 30 minutes under standard tissue culture conditions; B cells in suspension were then removed and washed three times in cold phosphate buffered saline (pH 7.4) before downstream analysis. We note that *Ebf1 −/−* cells were derived from female embryonic E13.5 livers and *Rag2 −/−* null cells were derived from adult male bone marrow.

### ChIP-seq analysis

Chromatin immuno-precipitation (ChIP) of tri-methylated lysine 4 of histone H3 (H3K4me3) and acetylated lysine 9 and 14 of histone H3 (H3ac) followed by massively parallel sequencing (ChIP-seq) in *Ebf1 −/−* pre-pro-B cells and *Rag1 −/−* pro-B cells was performed in Lin et. al. [5] (GEO accession: GSE21978). These raw data were obtained from the European Nucleotide Archive (ENA) and we assigned generic fastq quality scores to all reads, as no quality score was included with the sequence reads. Raw reads were aligned to the mouse reference genome (mm9) with Bowtie [20]. To generate predicted ChIP peaks, the MACS (Model-based Analysis of ChIP-Seq) algorithm was run on all samples (full parameter settings in Supplemental file 1) [21]. Peaks predicted for H3K4me3 and H3ac by MACS were merged for each cell line to demarcate ‘active regions’. Genes were predicted to have ‘active’ promoters as scored by MACS if a peak in the respective cell type overlapped by 10 base pairs (bp) a protein-coding RefSeq promoter, here defined by the 200 bp region upstream of the transcription start site (TSS).

As an alternative to MACS-based peak calling, to quantify the abundance of active chromatin modifications in discrete regions, reads of each ChIP-seq sample that showed any overlap with an interval as defined below were counted. These counts were normalized to the interval size and ChIP-seq library size, to generate a ChIP reads per kilobase per million reads value. H3K4me3 and H3ac ChIP reads per kilobase per million read values for each cellular state were summed to yield a cumulative ChIP reads per kilobase per million reads, which we refer to as the ‘cRPKM’ score. We calculated cRPKM for promoter regions, identified as 200 bp upstream and downstream of each annotated murine protein-coding RefSeq TSS (cRPKM_P_). For comparison, we further examined intervals of 400 bp from regions 2 kilobases (kb) upstream of TSSs (cRPKM_U_) and from 1 million random genomic intervals (cRPKM_R_). All ChIP-seq analysis was performed on Galaxy Cloudman using Amazon web services (AWS) [22,23].

### GRO-seq analysis

Global run-on sequencing was performed on *E2a −/−* pre-pro-B cells and *Rag1 −/−* pro-B cells in Lin et al. [17] (GEO accession: GSE40173). Corresponding raw data were obtained from ENA in fastq format, then cleaned by ‘Fastq quality trimmer by sliding window’ to trim both ends of the reads examining a window size of 3 nucleotides (nt), with a step size of 1 nucleotide, requiring ‘aggregates’ (windows) to meet a mean quality score greater than 20.0 for inclusion in the ‘cleaned’ data sets [24]. To filter reads of repetitive genes, cleaned GRO-seq reads were aligned to a custom genome consisting of all rRNA sequences (including immature) and the most canonical entry of each repetitive element in Repeat Masker (based on the Smith Watterman alignment score) with Bowtie [20]. Unmapped reads were mapped to a second custom genome consisting of the whole gene sequences of all mouse protein-coding RefSeq genes. Mapped reads were quantified as in [25] to generate reads per kilobase per million reads (RPKM) expression values for all protein-coding RefSeq genes. All differential RPKM expression values are expressed as log_10_(RPKM_pro-B_/RPKM_pre-pro-B_). All GRO-seq data analysis was performed with Galaxy Cloudman using AWS [22,23]. *E2a −/−* progenitor cells were derived from female embryonic E13.5 livers and *Rag1 −/−* pro-B cells were derived from male adult bone marrow.

### RNA-seq analysis

Total RNA was extracted from *Ebf1 −/−* pre-pro-B cells and *Rag2 −/−* pro-B cells by TRIzol, then subject to DNAse digestion to remove residual DNA and cleaned over a RNeasy (Qiagen 74104) column using the manufacturer’s ‘cleanup protocol’ to remove contaminants and deplete RNAs < 200 nt. RNA quality was assessed using a RNA 6000 nano total RNA kit (Agilent 5067-1511) with a 2100 Bioanalyzer (Agilent G2938C); all samples had RNA integrity numbers of 9.9. RNA samples were depleted of mature ribosomal RNA (rRNA) transcripts with Ribo ZERO (Epicentre RZH1064) following the manufacturer’s recommendation, using 2 µg of RNA as input. RNA depleted of rRNA was converted to indexed, strand-specific RNA sequencing libraries using the ScriptSeqv2 system (Epicentre SSV21106) following the manufacturer’s recommendation, using 50 ng of rRNA-depleted RNA as input. These RNA-seq libraries were sequenced on a HiSeq 2000 (Illumina SY-401–1001) to a read depth of ~90,000,000 single end 97 bp reads per sample. Raw reads were ‘cleaned’ using ‘Fastq quality trimmer by sliding window’ as described above. Cleaned reads were aligned to the mouse reference genome (mm9) with TopHat using strand-specific parameters [26]. Transcript abundance (measured as fragments per kilobase per million reads, FPKM) and differential expression estimates were generated by running Cuffdiff2.1.1 on the aligned reads using the protein-coding genes of RefSeq as the reference. RNA-seq analysis parameters are listed in Supplemental file 1 [27]. All differential FPKM expression values are expressed as log_10_(FPKM_pro-B_/FPKM_pre-pro-B_). All RNA-seq analysis was performed on Galaxy Cloudman using AWS [22,23]. FPKM/RPKM ratios were generated for each protein-coding RefSeq gene by dividing FPKM values of RNA-seq by RPKM values of GRO-seq.

### iTRAQ-based quantification of differential protein abundance

*Ebf1 −/−* pre-pro-B cells and *Rag2 −/−* pro-B cells were harvested as above, lysed in 0.5% SDS supplemented with complete EDTA-free protease inhibitors (Roche 11836170001) on ice, sonicated at 55 watts for 10 seconds for 5 cycles with an Ultrasonic Processor (GE601) at 4°C; samples were placed on ice for 1 minute between cycles. Lysates were clarified by centrifugation at 16,000 x *g* for 20 minutes at 4°C; then protein concentrations were determined using the BCA concentration assay (Pierce 23227), and samples were subsequently processed for iTRAQ analysis with the iTRAQ Reagents Multiplex Kit (AB Sciex 4352135) following the manufacturer’s recommendations with minor modifications. Briefly, 100 µg of each protein lysate was diluted to equal volumes with 0.5 M triethylammonium (dissolution buffer), then reduced using 3 mM tris-(2-carboxyethyl)-phosphine (TCEP, Reducing reagent) for one hour at 60°C and alkylated with iodoacetamide at a final concentration of 5.7 mM for 10 minutes at room temperature. Samples were diluted with 0.5 M triethylammonium to reduce SDS to < 0.05% and then digested with 1 µg Lys-C (Wako Chemicals USA, Inc.125-05061) for four hours at 37°C. Lys-C was inactivated by heating the digested peptides for 30 minutes at 60°C. Peptides were further digested with 1 µg trypsin (Promega V5111) for 12 hours at 37°C, then concentrated in a cooled speed vacuum to a volume of ~5 µL and diluted to 30 µL with 0.5 M triethylammonium. 3 µL of each peptide sample was removed to verify protein digestion by SDS-PAGE. The remaining 27 µL of the pre-pro-B and pro-B samples were labeled with iTRAQ 114 and iTRAQ 115, respectively. The labeled peptides from the two samples were mixed in equal amounts and diluted in 5 mM potassium phosphate buffer, 25% acetonitrile (pH 2.7) (solvent A). The labeled peptides were fractionated by offline strong cation exchange chromatography in the first dimension using a polysulfoethyl A column (5 µm, 200 × 2.1 mm, PolyLC) on an Agilent 1100 series HPLC system using a gradient of increasing salt concentration of up to 350 mM KCl in solvent A. A total of 9 fractions were prepared, dried in a vacuum dryer, reconstituted in 40 µL of 0.1% trifluoroacetic acid and desalted by STAGE-tips. Desalted peptides were loaded onto a trap column (2 cm, 3 µm size, 100 Å pore), separated in the second dimension by reversed phase liquid chromatography on an analytical column (15 cm, 3 µm, 100 Å) using Easy-nLC system (Thermo Scientific) and analyzed on a LTQ-Orbitrap Elite mass spectrometer (Thermo Scientific). The peptide ions were generated by spraying through a nanoelectrospray emitter tip (New Objective). The column contained 0.1% formic acid (solvent A) and was developed with solvent A mixed with the following steps or linear gradients of 90% acetonitrile, 0.1% formic acid (solvent B): 5% of solvent B for 2 min, 5–10% of solvent B for 2 min, 10–30% of solvent B for 70 min, 30–95% of solvent B for 11 min and 95% of solvent B for 17 min, all at a constant flow rate of 250 nL/min. The nanoelectrospray voltage used was 1.8 kV. Tandem mass spectrometry data were acquired in a data dependent manner. Briefly, the 15 most intense precursor ions from a precursor scan were selected for sequencing. Both MS and MS/MS scans were acquired using the *high-high* mode on an Orbitrap mass analyzer at resolution settings of 120,000 and 30,000, respectively. Higher energy collision dissociation technology was employed to fragment peptide ions with normalized collision energy of 35. The automatic gain control for full MS was set to 1,000,000 ions while for MS/MS it was set to 50,000 ions with maximum accumulation times of 100 msec and 300 msec, respectively.

The tandem mass spectrometry data obtained were searched against the mouse RefSeq 52 protein database using the Mascot database searching algorithm via Proteome Discoverer platform (version 1.4, Thermo Fischer Scientific). Search parameters were set as following: trypsin as the digestion enzyme allowing up to one missed cleavage; carbamidomethylation at cysteine and iTRAQ labeling at the N-terminus and lysine as a static modification; oxidation at methionine, protein N-terminal acetylation and pyroglutamine modification at glutamine as variable modifications; mass tolerance of 10 ppm for precursor ions and 0.8 Da for fragment ions. The cut-off of 1% false discovery rates was applied to peptide identification. Peptides unambiguously mapped to a single RefSeq gene were used for downstream integrative analysis.

## Results

### Examining the ability of chromatin modifications to predict changes in protein abundance – initial approach

To experimentally explore the power of differentially active chromatin modifications at promoters (which we will henceforth call predicted ‘active’ promoters) to predict differential protein levels from that gene, we first compared previously published chromatin data with proteomic data generated in our laboratory. Specifically, differential protein abundance between pre-pro-B and pro-B cells was determined using iTRAQ-based proteome quantification, a mass spectrometry technique that monitors relative changes in protein abundance across divergent cell types [28]. The resulting peptides were mapped to the RefSeq database to identify their cognate genes, enabling multi-omics analysis with genome-wide data. In addition, we used H3K4me3 and H3ac ChIP-seq data from pre-pro-B (*Ebf1 −/−* or ‘*Ebf1* null’) and pro-B (*Rag1 −/−* or ‘*Rag1* null’) cell lines (GEO accession: GSE21978) [5]. Initially, we employed the MACS peak calling strategy for identifying regions enriched in H3K4me3 and H3ac in both pre-pro-B and pro-B cells (see Methods). The identified peaks were then intersected with a database of murine protein-coding RefSeq promoters. A promoter was predicted to be ‘active’ if either a H3K4me3 or a H3ac peak overlapped the promoter.

**Figure 2:**
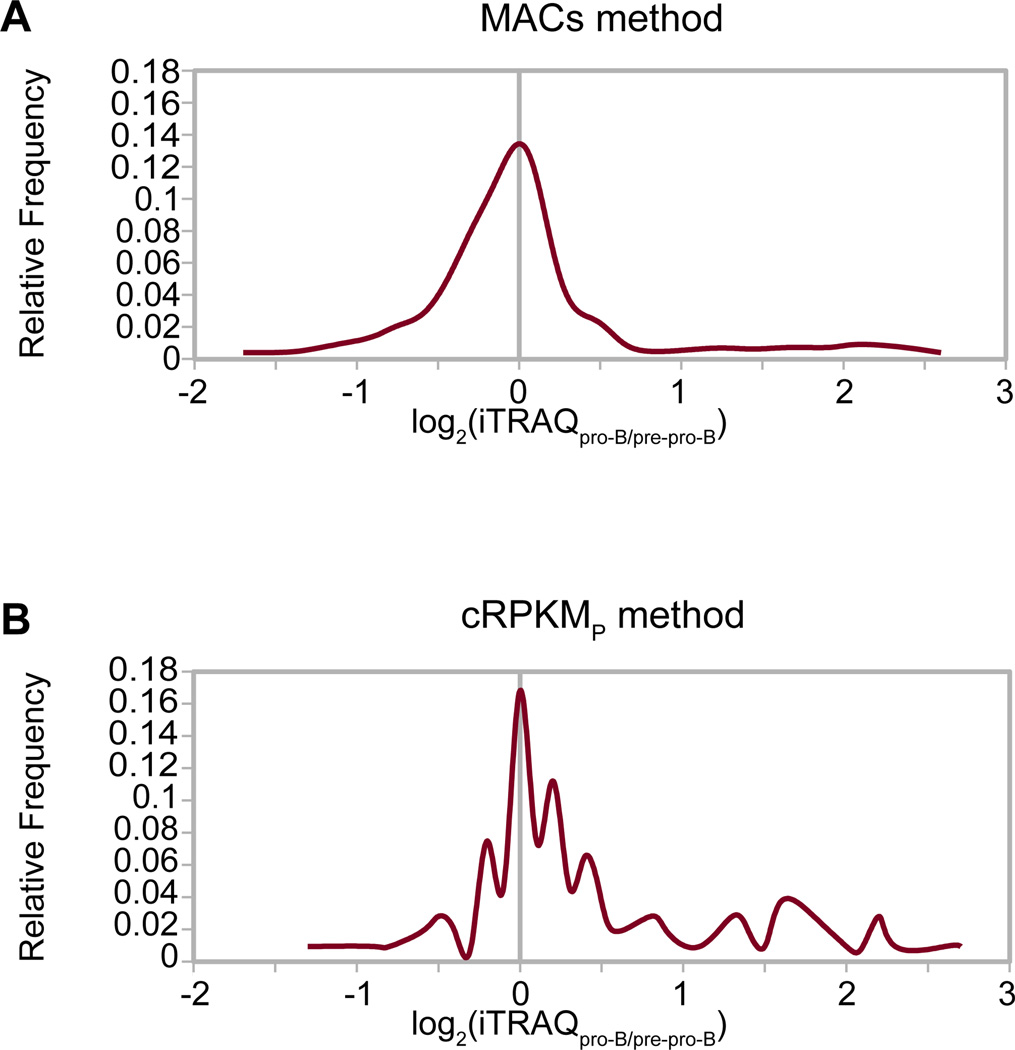
Comparison of MACS and cRPKM_P_ based chromatin prediction methods Directly comparing differential expression of proteins from genes predicted to be exclusively active in pro-B cell by the (A) MACS and (B) cRPKM_P_ (cut-off > 8) methods. The Y-axis represents the frequency in the population (‘relative frequency’) and the X-axis represents the log_2_(iTRAQ_pro-B/pre-pro-B_) of our iTRAQ-based proteome quantification data.

As a first step in determining how closely changes in promoter chromatin state correlated with protein differential abundance, we identified predicted differentially active promoters between pre-pro-B and pro-B cell stages. ‘Exclusively pro-B’ promoters have a peak of either or both of the active chromatin modifications overlapping the promoter region in pro-B cells but not in pre-pro-B cells. We then examined whether a substantial portion of the proteins corresponding to the ‘exclusively pro-B’ active promoters were differentially abundant by iTRAQ-based proteome quantification. Plotting the differential protein abundance [log_2_(iTRAQ_pro-B/pre-pro-B_)] as a frequency graph shows a general lack of concordance between promoters predicted by the MACS algorithm to be in the ‘exclusively pro-B’ active class and higher levels of their proteins in pro-B cells (Figure 2A). We had expected that these proteins would show a sizable population of positive differential abundance (i.e. skew to the right) indicating that many would be up-regulated in pro-B cells (Figure 2A). There was a similar lack of concordance between promoters predicted to be ‘exclusively pre-pro-B’ active class and higher levels of their proteins in pre-pro-B cells (data not shown).

This discordance led us to revisit our analyses of the ChIP-seq data, but we were unable to find parameters for the MACS algorithm that successfully discriminated many promoters as active, even though visual inspection clearly showed they had peaks in both chromatin modifications (Supplemental Figure 1, black bars). MACS missed even intense signals when they were in broad peaks, and further modifying our parameters resulted in a large number of false positives (Supplemental Figure 1 and data not shown).

### Examining the ability of chromatin modifications to predict changes in protein abundance in early B cell development – a modified approach

Given these issues with the MACS algorithm, we asked if we could use RPKM (reads per kilobase per million reads), similar to methods employed in quantifying RNA-seq data, to categorize promoters into ‘active’ versus ‘inactive’ based on their cumulative H3K4me3 and H3ac modifications. We postulated that by using a defined promoter region for each gene and not requiring a ‘peak’ to have a particular shape, this approach would not suffer from some of the issues we encountered when using MACS to identify promoters enriched in these active chromatin modifications. We identified promoters as within 200 bp (+/−200 bp) of the transcription start site (TSS) of all protein-coding RefSeq genes and then counted the number of reads for H3K4me3 and H3ac in the ChIP-seq data that overlap these regions. Summing the values for both active chromatin modifications provides what we are calling the cumulative ChIP RPKM (cRPKM, see Methods), a cumulative index of predicted promoter activity. Its cumulative nature should improve analyses since we need to overcome sequencing depth issues as we note that the sequencing depths of the previously published H3K4me3 and H3ac ChIP-seq libraries were 70% lower and 17% lower, respectively, in pre-pro-B cells than in pro-B cells (library depth can be found in Supplemental File 2 and in reference [5]).

As an initial attempt to validate the cRPKM method, we determined the distribution of cRPKM values for promoter regions of all annotated protein-coding genes in RefSeq (cRPKM_P_) in both pre-pro-B and pro-B cells, and compared that to the distribution of cRPKM values for corresponding regions 2 kb upstream (cRPKM_U_) and for randomly generated 400 bp genomic intervals (cRPKM_R_) (Supplemental Figure 2A and B). These data demonstrate that a substantial proportion of promoters display higher cRPKM distribution than regions 2 kb upstream or random genomic regions, which uniformly displayed relatively low cRPKM scores (Supplemental Figure 2A and B). Notably, the distribution of cRPKM_P_ values of promoters of all proteincoding RefSeq genes exhibits a clear separation into sub-populations displaying low and high cRPKM_P_ scores in both pre-pro-B and pro-B cells (Supplemental Figure 2A). Since only a fraction of all promoters would be expected to be active in pre-pro-B or pro-B cells, and since the distribution of the low cRPKM_P_ scores of promoters reflects the distribution of cRPKM_U_ and cRPKM_R_ scores of regions not selected to be promoters, we infer that this low cRPKM_P_ sub-population of promoters corresponds to ones that are inactive in this cell type. Conversely, the high cRPKM_P_ sub-population corresponds to the active promoters. As shown in the plot in Supplemental Figure 2A (see arrow), a cRPKM value of 8 discriminates these distinct populations of active from inactive promoters.

To validate our cRPKM_P_ of > 8 cut-off for active promoters we calculated cRPKM_R_ for one million random 400 bp genomic intervals irrespective of RefSeq promoter location and determined if those with cRPKM_R_ values > 8 selectively identified promoter regions (Supplemental Figure 3). This was expected because H3K4me3 and H3ac modifications have previously been demonstrated to be enriched on transcriptionally active promoters [29]. We employed the Genometricorr package [30], using relative distance tests to determine whether the random genomic intervals with cRPKM_R_ > 8 and with cRPKM_R_ < 8 are positioned closer to or further away from RefSeq TSSs than expected by chance. Both the Jaccard similarity coefficient and the projection statistical test (Genometricorr’s name for a binomial test of query positions compared to reference intervals) indicate that the random genomic intervals of cRPKM_R_ > 8 showed significantly more overlap with RefSeq promoters than expected by chance (Supplemental Figure 3A and B; p < 0.01), and conversely that the random genomic intervals of cRPKM_R_ < 8 were depleted in these regions (Supplemental Figure 3C and D; p < 0.01). Using the Genomic Hyper browser to perform analogous statistical tests yielded similar results (Supplemental Figure 3E) [31]. Given that these H3K4me3 and H3ac chromatin modifications reside on the 5’ region of active genes, the cRPKM_P_ > 8 cut-off predicts active promoters. Gratifyingly, this new cRPKM_P_ method to assess RefSeq protein-coding promoter regions is able to accurately capture representative active promoter elements that were missed using the MACS peak-calling algorithm (Supplemental Figure 1, gray bars). Thus, we will use cRPKM_P_ > 8 to predict active promoters for our subsequent main analyses.

**Figure 3:**
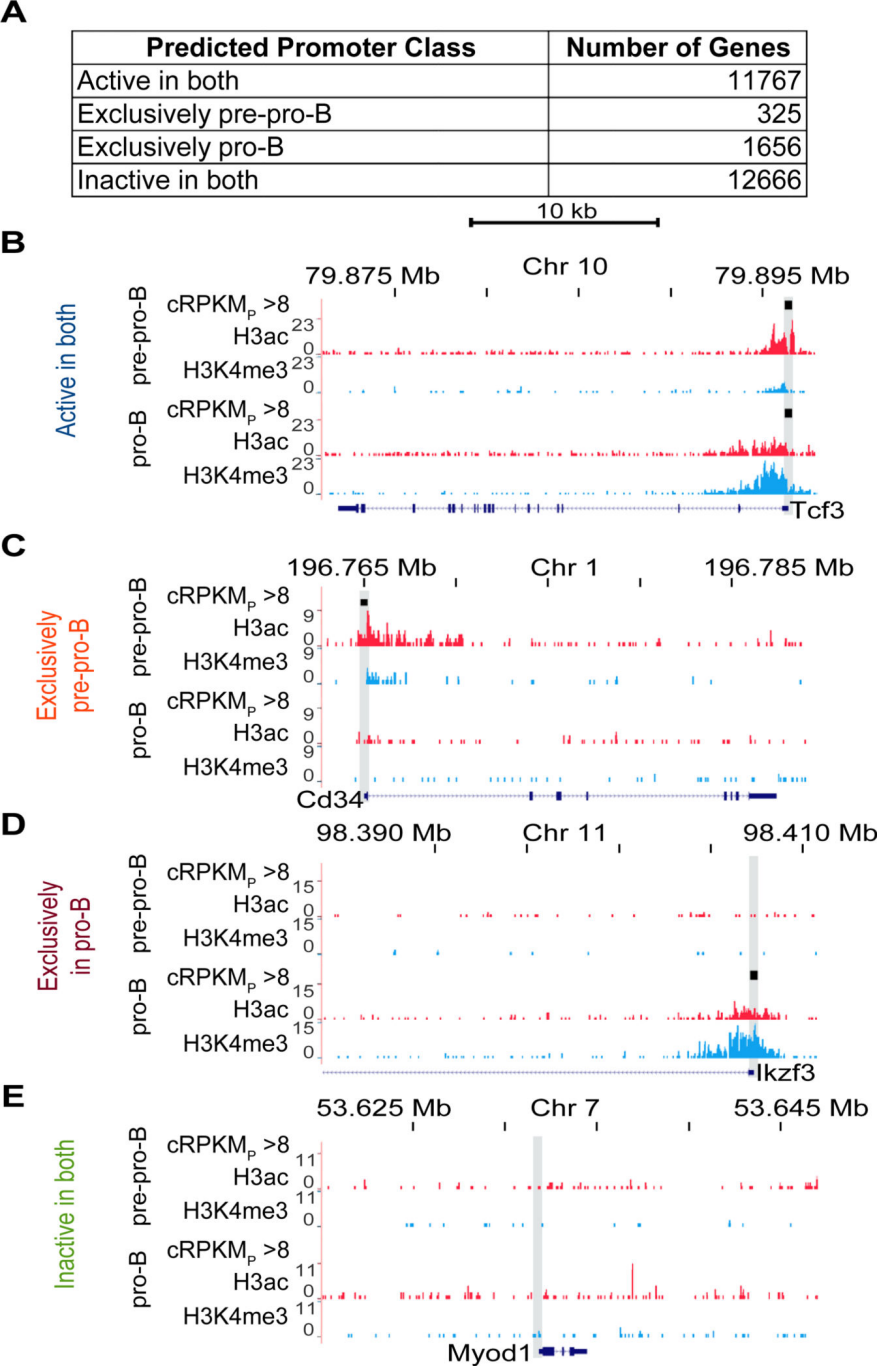
Representative genes in predicted promoter classes (A) Numbers of genes in the four predicted promoter classes: ‘active in both’, ‘exclusively pre-pro-B’, ‘exclusively pro-B’, and ‘inactive in both’. (B–E) An example of a gene in each of the four predicted promoter classes: Tcf3 (B), Cd34 (C), Ikzf3 (D), Myod1 (E). Each panel shows the H3ac (red) and H3K4me3 (blue) abundance for the indicated locus in each cell line. Promoters with cRPKM_P_ > 8 are indicated by black bars. Directional tick marks on the gene depictions indicate direction of transcription. The tall shaded boxes indicate the promoter regions of each gene. Genomic coordinates correspond to the most distal tick marks.

We next used the new cRPKM_P_ method with a cut-of of 8 to identify active or inactive promoters and grouped them into four classes according to their predicted differential activity between pre-pro-B and pro-B cells. The ‘active in both’ and ‘inactive in both’ classes represent a similar number of promoters (11,767 and 12,666, respectively) (Figure 3A). As expected, the exclusively active classes contain much lower numbers of promoters, with 325 identified as exclusively active in pre-pro-B cells (‘exclusively pre-pro-B’) and 1,656 as exclusively active in pro-B cells (‘exclusively pro-B’) (Figure 3A). However, those precise numbers might be affected by the depth of the ChIP-seq libraries, as addressed below. Very few genes displayed only H3K4me3 or only H3ac modifications on their promoters and all of these were the result of a single spurious amplicon skewing the number of reads observed, so those promoters were considered to be ‘inactive’. Importantly, this new cRPKM_P_ method correctly categorizes known differentially active genes, examples of which are shown in Figure 3B–E. As expected, the *E2a* promoter is classified as ‘active in both’, while the promoter of the muscle-specific *MyoD* gene [9] is classified as ‘inactive in both’ (Figure 3B and E). An example of a gene with a promoter representing the ‘exclusively pre-pro-B’ class is *Cd34*, which is known to be expressed in early hematopoiesis, including in multi-potent cells (Figure 3C) [32]. Also as expected, the developmentally regulated *Ikzf3* (Aiolos) promoter is in the ‘exclusively pro-B’ class, in agreement with previous data and its well documented transcription status during B cell development (Figure 3D and Figure 1) [33].

Using the now-verified cRPKM_P_ method to determine active promoters, we revisited our initial comparison of differentially active promoters with differential protein abundance. We again plotted the distribution of relative protein levels [log_2_(iTRAQ_pro-B/pre-pro-B_)] from promoters that were predicted to be exclusively active in pro-B cells (cRPKM_P_ method with a cut-off of 8, Figure 2B) and again we expected a substantial sub-population displaying higher protein abundance in pro-B than pre-pro-B cells (skewed to the right). This indeed was observed (Figure 2B), indicating that our cRPKM_P_ method yields a better correlation between predicted differential promoter activity and differential protein abundance. However, even using the cRPKM method to predict exclusively active pro-B promoters, an appreciable fraction does not display higher protein levels in pro-B cells (Figure 2B), contrary to expectation from a naïve strictly transcriptional regulatory model. An analogous comparison performed for genes with promoters predicted by cRPKM to be exclusively active in pre-pro-B cells also revealed an appreciable fraction that does not have higher protein levels in pre-pro-B cells (data not shown). Therefore, these populations indicate a lack of concordance between differential protein regulation and differential chromatin state. We next endeavored to investigate the coupling of chromatin regulation with the transcriptional and post-transcriptional steps of gene expression during these stages of B cell development by assaying the differential regulation occurring between pre-pro-B and pro-B cell stages by monitoring ongoing transcription (GRO-seq) through steady state mRNA levels (RNA-seq) and differential protein abundance (iTRAQ), relative to promoter chromatin status.

### cRPKM_P_ identified ‘active’ promoters identify stage specific transcriptionally active promoters

Since the predicted promoter classes have correctly identified key genes whose expression is known to be differentially regulated in the two different cell types (Figure 3) but do not fully correlate with differentially abundant proteins (Figure 2B), we asked how well these predicted promoter activity classes correlate with ongoing genome-wide transcription. To this end, we used publicly available global run-on-deep-sequencing (GRO-seq) data generated in murine pre-pro-B and pro-B cells (GEO accession: GSE40173) [17]. We aligned these strand-specific reads to a custom genome consisting of all gene loci that correspond to protein-coding RefSeq entries, and quantified the alignments to obtain reads per kilobase per millions reads (RPKM), as in [25]. We initially explored the relationship between cRPKM_P_ and RPKM by asking if our cRPKM_P_ cut-off of 8 delineates promoters that yield significant levels of transcription from those considered ‘inactive’, as would be expected. The cRPKM_P_ values for promoters of genes in the highest and with lowest quartiles of RPKM values were plotted against the extent of the RPKM value within that quartile, for pre-pro-B cells (Supplemental Figure 4A) and pro-B cells (Supplemental Figure 4B). As a group, the highly transcribed genes in the top quartile of GRO-seq values clearly have higher cRPKM_P_ values than the lowly transcribed genes in the bottom quartile (Supplemental Figure 4A and B), and the cRPKM_P_ cut-of of 8 is validated as it discriminates between these groups.

**Figure 4:**
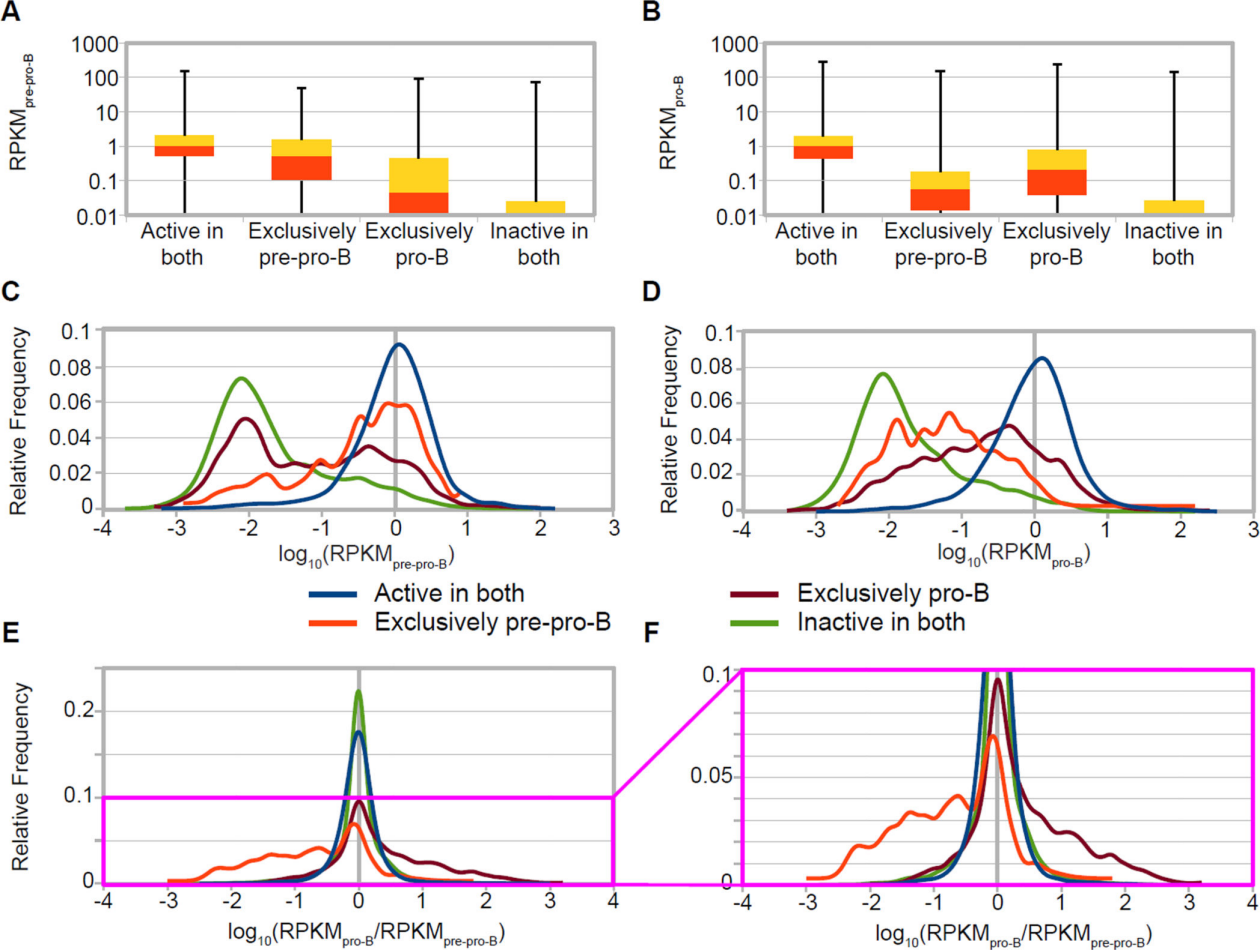
Relationship between predicted promoter class and ongoing transcription (GRO-seq) (A and B) Genes categorized based on their predicted promoter class (‘active in both’, ‘exclusively pre-pro-B’, ‘exclusively pro-B ‘, and ‘inactive in both’) are shown in box and whiskers plots representing the quartiles of GRO-seq values plotted versus GRO-seq transcription levels (RPKM on a log_10_ scale) in pre-pro-B cells (A) and pro-B cells (B). In these representations, all protein-coding genes in RefSeq are considered, including genes with RPKM = 0. (C and D) Graphs showing the relative frequency distribution of GRO-seq transcription levels [log_10_(RPKM)] for each predicted promoter class (see color key) in pre-pro-B cells (C) and pro-B cells (D). (E and F) Graphs showing the relative frequency distribution of the differential ratio of GRO-seq transcription levels [log_10_(RPKM_pro-B_/RPKM_pre-pro-B_)] for each predicted chromatin class, with the magenta box of (E) magnified in (F). In these representations, frequency was assessed at increments of 0.1 of the log_10_ converted transcription level (C and D) or calculated differential (E and F), and genes with RPKM = 0 are not included (C–F). All classes demonstrated statistically significant differences (WMW p < 0.0001).

We next examined the interrelationship between our promoter classes and ongoing active transcription. To enable such an analysis, we plotted RPKM values of the four predicted promoter classes (described above), both as quartile distributions in box-and-whisker plots (Figure 4A and B) and as frequency distributions of log_10_(RPKM) (Figure 4C and D). We would expect that genes with promoters in the predicted ‘exclusively pre-pro-B’ class would be enriched for high RPKM values in pre-pro-B cells (Figure 4A) but should not be enriched for high RPKM values in pro-B cells (Figure 4B). The converse should also be true for genes whose promoters are in the predicted ‘exclusively pro-B’ class. Also, genes with promoters in the ‘active in both’ and ‘inactive in both’ classes should, consistently in both cell types, display higher and lower RPKM values, respectively. We do indeed find that if a promoter is classified as either ‘active in both’ or ‘exclusively pre-pro-B’, that gene is much more likely to have a higher RPKM value by GRO-seq in pre-pro-B cells (Figure 4A and C). The same holds true for the ‘active in both’ and ‘exclusively pro-B’ classes in pro-B cells (Figure 4B and D).

In considering the above data, we note that the numbers of RefSeq genes represented in the ‘inactive in both’ (12,666) and ‘active in both’ (11,767) predicted promoter classes are substantially higher than the exclusively active classes (325 for pre-pro-B and 1,656 for pro-B), so the sum of the distribution of the GRO-seq values for ‘inactive in both’ and ‘active in both’ predicted promoter classes represents the general distribution of GRO-seq values. Given this, we note that the distribution of GRO-seq values form two quite distinct populations, one that is transcribed two to three orders of magnitude lower than the other and that our cRPKM_P_ > 8 cut-off discriminates between these two populations. In fact, we find that while approximately one quarter of the genes with predicted ‘inactive in both’ promoters had an RPKM of zero (and therefore are not represented in Figure 4C and D), the remaining three quarters did show expression, primarily at very low levels (Figure 4C and D). This expression may represent transcription in only a very small fraction of the cells at any one time, and when assayed by ChIP-seq, these promoters may well not have appreciable active chromatin modifications yielding cRPKM < 8 scores. Some of these RNAs could also reflect initiation from TSSs that were not annotated by RefSeq. In addition, while genes in both ‘exclusively active’ promoter classes have markedly different RPKM distributions between the two cell types, the distinctions between these classes are less pronounced than those between the ‘inactive in both’ and ‘active in both’ classes (Figure 4A–D). The cause of this discordance is unclear but could be due to many possible causes, including to a potential temporal discordance between alterations in active transcription and acquisition of promoter chromatin modifications. Statistical analyses using the Wilcoxen-Mann-Whitney test (WMW or U-test), a non-parametric analog of the t-test used in determining significance between skewed or non-normally distributed populations [33,34], revealed that all four predicted promoter classes were significantly different from each other, in both cell lines (p < 0.0001).

We found that the ‘active in both’ predicted promoter class contained not only numerous housekeeping and pleiotropic genes, as expected, but also genes previously shown as being involved B cell and early progenitor stage identity. For example, special AT-rich sequence binding protein 1 (*SatB1*), AT-rich interactive domain 3a (*Arid3a*), *Foxo1*, homeobox protein A3 (*Hoxa3*), and runt-related transcription factor 2 (*Runx2*), all factors implicated in hematopoiesis, are in this group, but show different levels of ongoing transcription [5,35–38]. Thus, it is important to note that some of these genes do display differential expression, even though they are in the ‘active in both’ class, indicating a co-transcriptional means of regulation uncoupled from changing promoter chromatin status as assayed by these two modifications (see later sections). In the exclusively active classes, by essentially filtering the GRO-seq data with predicted promoter status, we are able to identify and enrich for genes that are regulated at the transcription level coupled to a specific chromatin state change and implicated in B cell development. Examples of genes that are included in this group are Pituitary-specific Pit-1, Octamer transcription factor, and neural Unc-86 transcription factor class 2 associating factor 1 (*Pou2af1*), *Aiolos* (*Ikzf3*) and Interferon regulatory factor 4 (*Irf4*), which are upregulated in pro-B cells, and *Cd34*, which is up-regulated in pre-pro-B cells [1,13,32,33,39].

As expected, when plotting the frequency distributions of differential ongoing transcriptional activity [log_10_(RPKM_pro-B_/RPKM_pre-pro-B_)], the ‘exclusively pre-pro-B’ and ‘exclusively pro-B’ predicted promoter classes segregate, demonstrating that a large fraction of genes in these classes exhibit differential ongoing transcription levels (Figure 4E and F). The MACS peak calling strategy (shown in Supplemental Figure 5) does not predict ongoing transcriptional levels of the exclusively active predicted promoter classes as clearly as does the cRPKM_P_ method (compare especially Figure 4C and F with Supplemental Figure 5C and F).

### cRPKM_P_ identified ‘active’ promoters identify stage specific steady-state mRNAs

**Figure 5:**
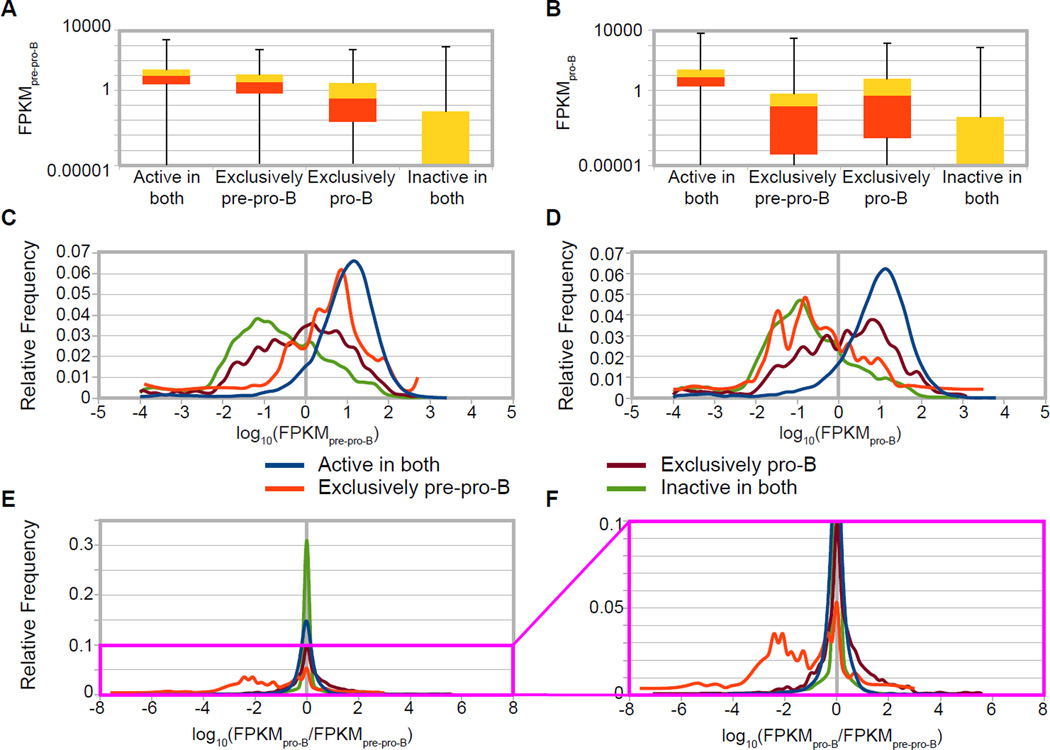
Relationship between predicted promoter classes and steady state mRNA levels (RNA-seq) (A and B) Genes categorized based on their predicted promoter class (‘active in both’, ‘exclusively pre-pro-B’, ‘exclusively pro-B’, and ‘inactive in both’) are shown in a box and whiskers plots representing the quartiles of RNA-seq values plotted versus RNA-seq transcription levels (FPKM on a log_10_ scale) in pre-pro-B cells (A) and pro-B cells (B). In these representations, all protein-coding genes in RefSeq are considered, including genes with FPKM = 0. (C and D) Graphs showing the relative frequency distribution of RNA-seq transcription levels [log_10_(FPKM)] for each predicted promoter class (see color key) in pre-pro-B cells (C) and pro-B cells (D). (E and F) Graphs showing the relative frequency distribution of the differential ratio of RNA-seq steady state mRNA levels [log_10_(FPKM_pro-B_/ FPKM_pre-pro-B_)] for each predicted chromatin class, with the magenta box of (E) magnified in (F). In these representations, frequency was assessed at increments of 0.1 of the log_10_ converted transcription level (C and D) or calculated differential (E and F), and genes with RPKM = 0 are not included (C–F). All classes demonstrated statistically significant differences (WMW p <0.0001).

Since ongoing transcription was expected to be highly correlated with promoter status and differential transcription with differential promoter status, we next asked if these general trends hold true when comparing the various predicted promoter classes with steady state RNA levels (Figure 5). To enable such an experimental test, we generated genome-wide RNA-seq data in our model system. Specifically, isolated total RNA from *Ebf1* null pre-pro-B cells and *Rag2* null pro-B cells was depleted of ribosomal and small RNAs using RiboZero and size exclusion columns, respectively. This mRNA-enriched preparation was subjected to standard RNA-seq library preparation and sequenced on an Illumina HiSeq 2000. Sequence reads were aligned with TopHat, quantitative estimation of FPKM (fragments per kilobase per million reads) was assigned to each protein-coding RefSeq gene for each cell type, and differential expression was determined using Cuffdiff2.1.1 [26,27]. Not surprisingly, these steady state RNA levels demonstrate a general concordance with expected gene activity based upon predicted promoter classes (Figure 5A–D). The distribution of FPKM values for the different predicted promoter classes demonstrates higher steady state mRNA levels for active promoters, whether ‘active in both’ or ‘exclusively’ active in the appropriate cell type. Thus, the ‘exclusively pre-pro-B’ predicted promoter class displays higher FPKM levels than the ‘exclusively pro-B’ class in pre-pro-B cells, and vice versa in pro-B cells. However, the differences between these ‘exclusive’ classes appear less substantial, although statistically significant, than the different distributions of abundance between the ‘active in both’ and ‘inactive in both’ predicted promoter classes (Figure 5A–D), as was also observed in the GRO-seq analysis (WMW p < 0.0001) (Figure 4). Only one third of the genes in the predicted ‘inactive in both’ predicted promoter class yielded transcripts with detectable RNA by RNA-seq (FPKM > 0), consistent with their inactive chromatin status, and the majority of these transcripts displayed several orders of magnitude lower RNA abundance than genes in the ‘active in both’ predicted promoter class. As expected, the exclusively active promoter classes display global differential steady-state mRNA expression levels [log_10_(FPKM_pro-B_/ FPKM_pre-pro-B_)] (Figure 5E and F). The MACS strategy was employed to predict promoter classes (Supplemental Figure 6) and was much less successful at faithfully classifying differentially active promoters (compare Figure 5C and F with Supplemental Figure 6C and F).

Within the cRPKM_P_ defined ‘active in both’ predicted promoter class some genes do display differential steady state RNA levels between pre-pro-B cells and pre-B cells. As indicated above, numerous genes also displayed differential ongoing transcription (GRO-seq), thus there are likely co-transcriptional and post-transcriptional means of regulating mRNA levels (e.g. RNA stability, see below). In the exclusively active classes, by filtering the RNA-seq data with predicted promoter status, we are able to identify and enrich for genes that are likely mostly transcriptionally regulated in a specific developmental program (i.e. at the level of chromatin—active in both-, but differential ongoing transcription and differentially maintained steady-state RNA levels). Examples of known B cell development regulatory genes that are included in this group are *Pou2af1*, *Aiolos* (Ikzf3), and *Irf4* that are upregulated in pro-B cells, while *Cd34* and *Flt3* are upregulated in pre-pro-B cells, most of which were also identified as displaying differential ongoing transcription (GRO-seq) [1,6,13,32,33]. However, there are a number of genes that appear to have differential mRNA abundance that cannot be ascribed to differential levels of ongoing transcription (see below).

### A subset of genes with ‘active’ promoters are regulated by RNA stability

In order to understand the role that RNA stability may play in regulating early B-cell development, we next compared our GRO-seq with our steady-state RNA-seq data for all expressed genes. Specifically, we plotted RPKM (ongoing transcription) versus FPKM (steady state mRNA) for both cell types, scoring each promoter class separately as well as all genes together (Supplemental Figure 7). These data were divided into quadrants to more easily separate active and relatively inactive genes. As expected, genes with promoters in the predicted ‘active in both’ class display the densest distribution in the upper right quadrant for both cell types, i.e., high RPKM and high FPKM values (Supplemental Figure 7A and B). Conversely, genes with promoters in the predicted ‘inactive in both’ class display the greatest density in the lower left quadrant, i.e., low RPKM and low FPKM (Supplemental Figure 7C and D), with the majority of the genes in this promoter class not represented on these plots as they did not have measurable RPKM and/or FPKM values. For genes with promoters in the exclusively active predicted classes, one would expect the high density signal to be in the upper right quadrant (high RPKM and FPKM) for the cognate cell type and in the lower left quadrant for the alternate cell type (low RPKM and FPKM). This is indeed the case for genes with promoters in the predicted ‘exclusively pre-pro-B’ class (Supplemental Figure 7E and F) and for genes with promoters in the predicted ‘exclusively pro-B’ class in pro-B cells (Supplemental Figure 7H). However, genes with promoters in the predicted ‘exclusively pro-B’ class in pre-pro-B cells show a significant deviation from this pattern, with two obvious populations, not only the expected low RPKM and low FPKM population but also an appreciable amount of a high RPKM and high FPKM population (Supplemental Figure 7G). That population could potentially be explained if the lower ChIP-seq library depth in pre-pro-B cells, which was noted above, led to under-calling of promoters with activating chromatin modifications in this cell type. This would cause some promoters that should really be in the ‘active in both’ class to be mis-classified as ‘exclusively pro-B’. Nonetheless, there is a clear enrichment of differentially active genes based upon our predictions in both ‘exclusively pre-pro-B’ and ‘exclusively pro-B’ classes, compared to the distribution of all genes (Supplemental Figure 7I and J) and the ‘active in both’ and ‘inactive in both’ classes.

A small but obvious minority of the genes in each promoter class, but especially the ‘active in both’ predicted promoter class, have much lower FPKM (steady state RNA) than RPKM (ongoing transcription) levels (bottom portion of the graphs in Supplemental Figure 7A and B and highlighted in Supplemental Figure 8A and B). This suggests that the transcripts of these genes may be markedly unstable and that RNA stability of these genes could possibly play a significant role in early B cell development. To examine whether the genes displaying this apparent RNA instability are the same or different between the two cell types, we specifically compared their differential expression values in the two cell types by plotting the differential steady state mRNA abundance [log_2_(FPKM_pro-B_/FPKM_pre-pro-B_)] on the Y axis and differential ongoing transcription [log_2_(RPKM_pro-B_/RPKM_pre-pro-B_)] on the X axis, color-coding these data for the two cell types (Supplemental Figure 8C). There are three discrete sub-populations, with the majority of these genes exhibiting large positive or negative differential levels of steady state mRNA level between the two cell types but minimal differential levels of ongoing transcription. Color-coding the genes to indicate ones that are in this low-FPKM sub-population only in pre-pro-B cells (orange), only in pro-B cells (burgundy) or in both cell types (blue) shows that a large proportion of these genes exhibit this striking instability only in pre-pro-B cells or only in pro-B cells, although some genes exhibit the same differential in both cell types (Supplemental Figure 8C). Intriguingly, several genes encoding transcription factors and chromatin modifiers (along with many others) previously implicated in B cell development were identified to be amongst these differentially unstable mRNAs, including [(DNA methyltransferase 3a (*Dnmt3a*) and cyclic AMP responsive element binding protein 1 (*Creb1*)] [40,41]. These analyses also identified genes encoding many zinc finger proteins (Zfp) and zinc finger and BTB (Zbtb) transcription factors of unknown import to B cell development (*Zfp27*, *Zfp94*, *Zfp120*, *Zfp275*, *Zfp710*, *Zfp821*, *Zbtb3*, *Zbtb25*, *Zbtb43*, *Zbtb44*) as potentially being regulated post-transcriptionally through a mechanism that affected RNA stability (see supplemental file 3).

**Figure 6:**
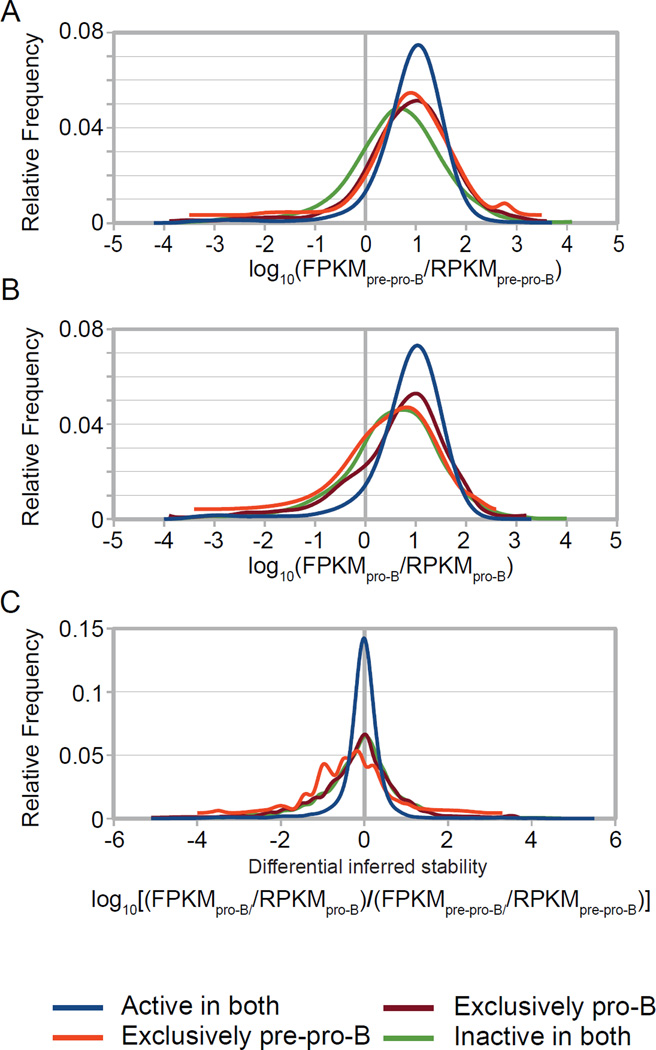
Relationship between predicted promoter classes and FPKM/RPKM ratio (A and B) Graphs showing the relative frequency distribution of FPKM/RPKM ratios [log_10_(FPKM/RPKM)] for each predicted promoter class (see color key) in pre-pro-B cells (A) and pro- B cells (B). Plot displaying the differential ratio of log_10_[(FPKM_pro-B_/RPKM_pro-B_)/(FPKM_pre-pro-B_/RPKM_pre-pro-B_)] for each predicted promoter class (C). Frequency was assessed at increments of 0.1 of the calculated ratio and genes with zero value for either RPKM or FPKM were excluded from this analysis (A–C). All classes demonstrated statistically significant differences, with the exception of ‘exclusively pre-pro-B’ and ‘active in both’ in pre-pro-B cells (WMW p < 0.0001).

Since we observed that certain populations of genes could have such dramatic cell type specific differential between steady state mRNA levels and ongoing transcription levels, we explored the global relationship between our predicted promoter classes and mRNA stability—specifically asking if promoter status may somehow be linked to this type of post-transcriptional regulation. By taking the ratio of FPKM (steady state mRNA) and RPKM (ongoing transcription) values for all protein-coding RefSeq genes, we generated an inferred measure of stability for each transcript. While the frequency distributions of these calculated ratios for the two exclusively active predicted promoter class appears quite similar in both cellular state (Figure 6A and B), and when compared between cellular states (Figure 6C), statistical analyses reveal that these actually represent different populations (WMW p < 0.0001), with the ranked means of the active classes being larger than the inactive classes (i.e. active groups appear to generate more stable mRNAs). Thus, it is tempting to speculate that the modifications on active promoters (in this case H3K4me3 and H3ac) not only affect the transcriptional on/off state, but also transcript stability, perhaps through their reported role in facilitating elongation [42]. This same analysis performed with the MACS-based promoter prediction strategy showed no striking global differences (Supplemental Figure 9). Taken together, these data show that a number of genes display differential stability in a developmental stage specific manner.

### The majority of differentially abundant proteins do not derive from differentially active promoters

**Figure 7:**
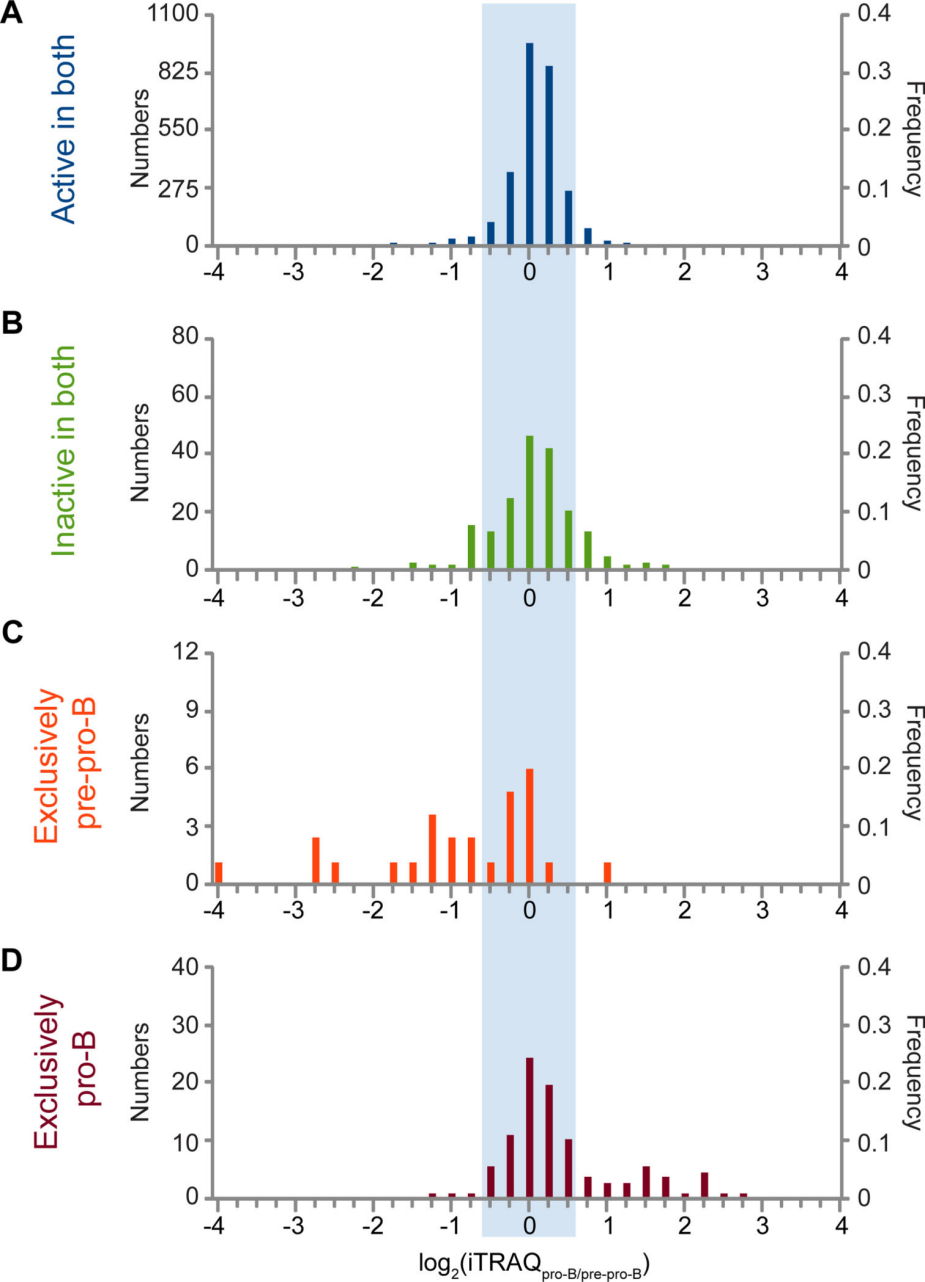
Relationship between predicted promoter classes and protein differential abundance Histograms of differential protein abundance [log_2_(iTRAQ_pro-B/pre-pro-B_)] for genes of the ‘active in both’ (A), ‘inactive in both’ (B), ‘exclusively pre-pro-B’ (C), and ‘exclusively pro-B’ (D) predicted promoter classes. The shaded region of the plot represents non-differentially abundant proteins and the unshaded region indicates differentially abundant proteins based on a 1.5 fold difference threshold. Each bar on the plots represents a bin with a range of 0.25.

We next returned our focus to the relationship of the predicted promoter classes with differential protein abundance and, thus, regulation of B cell development. The above mentioned iTRAQ-based proteome quantification from pre-pro-B cells and pro-B cells generated in our laboratory yielded 27,662 peptides which mapped to 3,129 unique proteins. While most proteins displayed no differential abundance, we identified 337 differentially abundant proteins between the cell types (based on +/−1.5 fold change) (Figure 7A–D, unshaded area). A number of these agree with previously published results, verifying our analyses and appropriate differential expression of these genes in our model system. *Ikzf3* (Aiolos), *Pou2af* and *Irf4* , genes previously shown to be up-regulated in pro-B cells, are in our ‘exclusively pro-B’ predicted promoter class and are up-regulated in our proteome data (Figure 3, Supplemental File 3) [33,39,42,43]. Also, *Cd34* and *Flt3*, well-established to be down-regulated upon progression through the hematopoietic differentiation program [32], are in our ‘exclusively pre-pro-B’ predicted promoter class (Figure 3, Supplemental Data File 3) and are down-regulated in our proteome data. Interestingly, our data identify a number of additional proteins that display lower abundance levels at the pro-B cell than pre-pro-B cell stage, consistent with a role in the earliest stages of B cell specification. One example is Cst7 (leukocystatin), which is known to regulate effector serine proteases in thymocytes (T cells) and natural killer cells (NK cells) [44], and we find is ~7 fold down-regulated in pro-B cells at the protein level. Since pre-pro-B cells are a common lymphoid progenitor with the capacity to differentiate towards many immune cell lineages, Cst7 may be in a poised state in pre-pro-B cells, to be up-regulated if the differentiation cascade follows a T cell or NK cell pathway or down-regulated if the differentiation cascade proceeds down the B cell pathway.

Given the mass spectrometry methodology and strategy we employed, we were able to perform integrated analyses on only a subset of the genes and promoters used in our previous analyses (with all identified peptides mapping to 3,129 RefSeq genes, versus 26,414 genes assayed by ChIP-seq, GRO-seq and RNA-seq). The largest number of iTRAQ identified proteins come from genes with unchanging predicted promoter classes, almost all of these are from the predicted ‘active in both’ promoter class (2,808 proteins). However, a few are from the predicted ‘inactive in both’ class (189 proteins); they could derive from gene products with TSSs that are not annotated, genes that are only active in a very small fraction of the cells or for a very small fraction of the time but give protein products that yield peptides that are favored for detection by our LC-MS/MS strategy, or from genes with mis-classified promoters. As expected, the vast majority of the proteins encoded by the genes in the ‘active in both’ and ‘inactive in both’ classes do not have significant differential abundance values (Figure 7A and B, shaded area). Conversely, when focusing on the proteins from the predicted exclusively active promoter classes, a substantial number display differential abundance (Figure 7C and D). This is especially striking for the ‘exclusively pre-pro-B’ class (Figure 7C); the data for the ‘exclusively pro-B’ class (Figure 7D) may appear less dramatic because the low ChIP-seq library depth for the pre-pro-B cells could cause this predicted chromatin class to be contaminated with promoters that are truly ‘active in both’, as noted above. Nonetheless, the majority of the differentially abundant proteins do not appear to come from promoters that are predicted to be differentially active by our cRPKM_P_ method, suggesting post-chromatin regulation, and likely post-transcriptional regulation. The MACS-based prediction of exclusively active chromatin classes performs poorly in enriching for differentially abundant proteins (Figure 2A and Supplemental Figure 10).

### Multi-omics analyses reveal that differential protein abundance in early B cell development is predominantly due to post-transcriptional regulatory mechanisms

We further considered the inter-relationships between predicted promoter classes and differential transcription, transcript steady state, and protein levels. For these mutli-omics differential analyses, there are 27 possible regulatory combinations of differential abundance at the ongoing transcription, steady state mRNA, and protein levels. Defining differential abundance as a change of at least 2-fold, 2-fold, and 1.5-fold, respectively, Supplemental Table 1 shows the numbers and percent of genes that are within each of these regulatory combinations, for all genes and for each of the four predicted chromatin classes. Due to the limited dynamic range of our quantitative proteomic data, we were not able to use the classical 2-fold cut-off to define the most differentially abundant — even using a less-stringent cut-off of 1.5-fold resulted in limiting these analyses to the top 7–15% differentially expressed proteins. A large fraction of genes with predicted exclusively active promoters exhibit corresponding changes at the ongoing transcription, steady state mRNA, and protein levels (Supplemental Table 1). Furthermore, the vast majority of genes with predicted ‘active in both’ promoters do not exhibit differential ongoing transcription, steady state mRNA, or protein levels (Supplemental Table 1). While this analysis is certainly a broad view, and the precise placement of genes into the regulatory combinations is in part subject to the precise cutoffs for differential abundance, a significant fraction of genes appear to display potentially unexpected regulatory combinations with differential RNA expression but not substantially differential protein levels as detected by iTRAQ. These remain to be further investigated. To further interpret and summarize these data, we established broader regulatory groupings: 1) differential protein abundance (iTRAQ) with supporting differential transcription (GRO-seq) and steady state mRNA levels (RNA-seq), 2) differential protein abundance evidently due to post-transcriptional mechanisms (no change in ongoing transcription (GRO-seq), irrespective of mRNA steady-state levels), 3) undetermined or ambiguous inter-relationships, and 4) no called changes in protein abundance, irrespective of the ongoing transcriptional and steady state mRNA data (Supplemental Table 1). Table 1 summarizes the percent of genes that fall into these broader regulatory groupings. Strikingly, for genes displaying high confidence differential protein abundance, not parsed out by predicted promoter class, over half appear regulated predominantly by post-transcriptional mechanisms (Table 1, ‘DE genes’ 52.5%). Approximately 1/3 of them appear to be regulated at the steady state RNA level (possibly due to differential efficiencies of RNA maturation or differential RNA stability), while approximately 2/3 of them appear to be regulated at a subsequent step (possibly due to differential efficiencies of translation or differential protein stability) (Supplemental Table 1). Parsing out the genes that display high confidence differential protein abundance (above the 1.5 fold cut-off for protein differential) into predicted chromatin classes, post-transcriptional regulation is also the predominant regulatory mode in the ‘active in both’ and ‘inactive in both’ classes (58% and 54.9%, respectively) (Table 1), again about 1/3 at the steady state RNA level and 2/3 at a subsequent step (Supplemental Table 1). In contrast, the vast majority of the genes in the ‘exclusively pre-pro-B’ and ‘exclusively pro-B’ classes with differentially abundant proteins follow a transcriptional regulatory mode (Table 1, Table 87% and 71%, respectively), as expected. We find these observations to be striking: that chromatin modifications leading to a direct transcription based model of differential protein expression account for less than half of the observed high confidence protein differential between these developmental stages.

We next asked whether transcriptional or post-transcriptional regulatory mechanisms yielded, in general, larger changes in protein levels in our developmental system. A box and whiskers plot indicates that the magnitude of differential expression was substantially larger for genes that are transcriptionally regulated than genes that are post-transcriptionally regulated when assessed at the steady state mRNA level, as expected (Supplemental Figure 11A), and apparently also larger when assessed at the protein level (WMW p < 0.0001) (Supplemental Figure 11B). Thus, while transcriptional regulatory mechanisms accounted for less than half the differential protein levels noted, regulation by post-transcriptional mechanisms yielded more genes with changes in protein levels. Further interrogating our multi-omics data set confirmed that many genes previously implicated in stage-specific expression patterns in our developmental model and mentioned above are regulated by transcriptional mechanisms, as expected (Supplemental Table 2). This is not surprising since networks of transcriptional regulation and changing chromatin state are clearly important for driving the B cell developmental program. However, we also found differentially abundant proteins that were regulated by either transcriptional or post-transcriptional mechanisms and that have not previously been implicated in our developmental model as shown in Supplemental Table 2 (and data not shown). Of these genes, *Galnt11* is of particular interest, as it has recently been shown to be up-regulated in chronic lymphocytic leukemia (CLL) and associated with poor disease prognosis [45].

**Figure 8:**
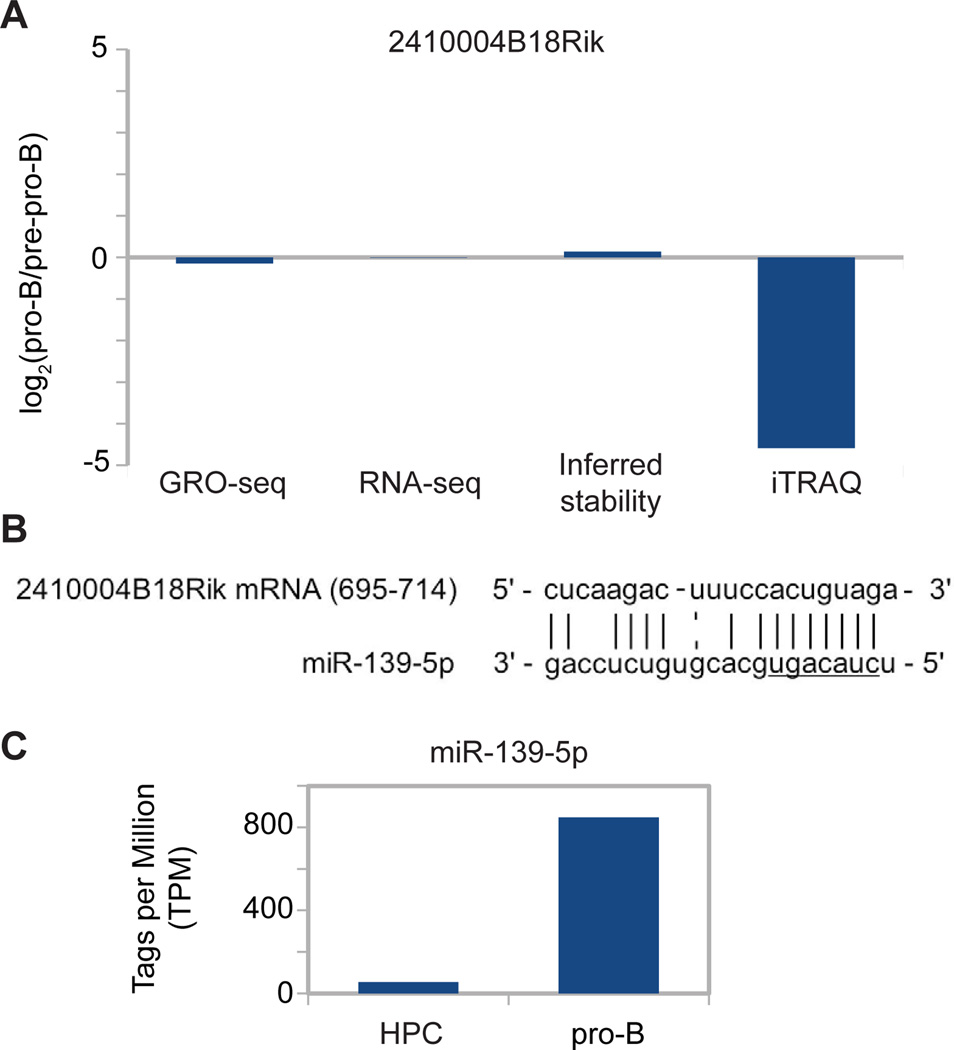
Post-transcriptional regulation of *2410004B18Rik* (A) Differential expression values at the ongoing transcriptional (GRO-seq), steady state mRNA (RNA-seq), inferred stability and protein levels of 241000B18Rik, represented as log_2_(pro-B/pre-pro-B) values. (B) Representation of the 3’ UTR of *2410004B18Rik*:miR-139-5p complementarity with the nucleotide positions of *2410004B18Rik* indicated and the miR-139-5p seed sequence underlined. (C) Mature miR-139-5p abundance estimates in hematopoietic progenitor cells (HPC) and pro-B cells [52].

Notably, the most dramatically differentially abundant protein observed in our developmental model is encoded by 2410004B18Rik (24-fold down regulated between pro-B and pre-pro-B cell states), even though this gene is in the ‘active in both’ predicted promoter class (Supplemental Figure 12, Supplemental Table 2). A preliminary literature search revealed no known function for 2410004B18Rik, although it appears evolutionarily conserved and is present in humans (C1orf52). While *in silico* protein functional analyses failed to predict known functional domains or sequence similarity with proteins of known structure or function (data not shown), querying the IntAct protein-protein interaction database revealed that 2410004B18Rik interacts with two important transcription factors implicated in immune pathways, NFκB (nuclear factor of kappa light polypeptide gene enhancer in B cells) and Ets-1 (E26 avian leukemia oncogene 1) [46]. Interestingly, *2410004B18Rik* displays no differential ongoing transcription (GRO-seq), steady-state RNA (RNA-seq), or inferred stability levels (Figure 8A), possibly suggesting regulation at the level of translation or protein stability. To investigate the regulatory mechanism(s) that mediate such a dramatic change in 2410004B18Rik protein levels, without affecting transcriptional and steady state mRNA levels, we explored the possibility of microRNA-mediated regulation. Published microRNA:mRNA target prediction databases (microRNA.org/miRanda, PITA, miRwalk, and miRTcat) revealed only one microRNA, miR-139-5p, is predicted by all four databases to have binding site(s) in the *2410004B18Rik* 3’ UTR (Figure 8B) [47–50]. Interestingly, miR-139-5p has previously been shown to regulate Foxo1 protein levels in hepatocytes without changing its mRNA levels [51]. Furthermore, mining of published microRNA deep sequencing data from murine lymphoid lineage cells shows that miR-139-5p is up-regulated 15.5 fold between pre-pro-B and pro-B cells (Figure 7C) [52]. Based on published evidence that miR-139-5p can regulate protein levels independent of corresponding mRNA transcript levels, predicted interactions of miR-139-5p with the 3’ UTR of *2410004B18Rik* by four miR: mRNA interaction databases, and an up-regulation of miR-139-5p that is reciprocal to 2410004B18Rik protein level down-regulation in pro-B cells relative to pre-pro-B cells, we propose that miR139-5p could act to down-regulate 2410004B18Rik protein levels independent of changes in transcript levels. This awaits further investigation.

## Discussion

Numerous studies have demonstrated that promoters enriched in H3K4me3 and H3ac are correlated with gene activity [29]. The majority of these studies have either focused on correlation with steady-state transcript levels, or more recently, utilized differential chromatin modifications as a proxy for identifying genes whose protein products may be regulated in and important for developmental progression [53,54]. To date, integrative studies demonstrating a direct link between changes in chromatin state during development and differential protein expression have been lacking [18,19]. Using existing and new data from an early B cell developmental model system, we addressed how gene expression is regulated from the chromatin state to the protein level and how these inter-relationships may affect B cell development.

In this systems biology approach, we determined whether gene expression is regulated by chromatin, ongoing transcription, transcript steady state, and/or post-transcriptionally, using differential protein levels as the end point measure of gene expression—the end point that actually is an effector of cellular state. We affirm that active chromatin modifications at promoters can in general be used to predict genes that are actively transcribed (Figure 4) and have significant steady state mRNA accumulation (Figure 5). We further show that developmentally differential promoter-proximal chromatin modifications also predict differential protein levels for an appreciable fraction of their assessed proteins (Figure 7C and D). However, we show that such predictions of activity from chromatin state miss many proteins that are differentially abundant, largely due to post-transcriptional regulation (Table 1; Supplemental Table 1). Indeed, our analysis confirms the striking finding that over half of all differentially abundant proteins appear to be regulated by some post-transcriptional mechanism (Table 1; Supplemental Table 1) [55,56]. However, the distribution between transcriptional and post-transcriptional regulation is dramatically different for the various predicted promoter classes. Although transcriptional regulation is the predominant mode to modulate protein abundance in genes whose promoter is predicted to be exclusively active in one of the cell types, we notably demonstrate that changes in protein levels from genes harboring non-differentially active promoters are largely post-transcriptional and therefore uncoupled from active chromatin state (Table 1). Evidently about 1/3 of those instances are regulated at mRNA steady-state abundance and about 2/3 at a subsequent stage (Supplemental Table 1). Thus, a change in chromatin state often predicted transcriptional and protein level changes, while changing protein levels did not indicate a change in promoter chromatin state. We identified many genes who display differential regulation that is uncoupled from their chromatin state, such as *SatB1*, *Arid3a*, and *Foxo1*, all genes involved in hematopoiesis [5,35,36]. In fact, the most up-regulated protein in our model system displayed no changes in its promoter status (Supplemental Figure 12). It is from gene 2410004B18Rik, which we show is regulated beyond the level of steady-state mRNA abundance, and our analyses further provide evidence that expression of this gene is regulated by miR-139-5p post-transcriptionally (Figure 8).

To enable these analyses, we introduce a new robust cRPKM_P_ method of analyzing ChIP-seq data (Figure 3) that appears more efficient at identifying active chromatin modifications at promoters, and thus at predicting gene activity, than is the MACS algorithm as we used it (Supplemental Figure 1). We showed that a number of genes that are implicated as hallmarks of our differentiation model agree with previous observations (Supplemental Table 2), and our collective integrated data can be mined at the gene level to uncover gene expression patterns that are involved in differentiation (e.g., Supplemental Table 1). Many leukemias are hypothesized to arise from early immune developmental stages, and further interrogation of our data could lead to a better understanding of the developmental mechanisms perturbed in such diseases. Moreover, with further advancement of proteomics tools, such as stable isotope labeling by amino acids in cell culture (SILAC) and iTRAQ, the next generation of such analyses will likely uncover more developmentally important proteins that are regulated post-transcriptionally and thus provide further examples where the dynamic epigenome is uncoupled from protein expression. Finally, we submit this type of data integration should be of great use in elucidating relationships between miRNAs and their potential targets on a genome-wide scale, as the GRO-seq data provide a means to rule out transcriptional regulation in modulation of mRNA levels.

## Supplementary Material

Supplemetary files

## Figures and Tables

**Table 1:** Mechanisms of differential protein abundance The percent of all genes (top) and of genes with protein differential abundance (‘DE’, bottom) that fall in the broad regulatory groups: ‘transcriptional’, ‘post-transcriptional’, ‘undetermined or ambiguous mechanism’, and ‘no protein change’, as determined in Supplemental Table 1.

	% All genes	% Active in both	% Exclusively pre-pro-B	% Exclusively pro-B	% Inactive in Both	
Transcriptional (1)	4.0	2.8	52.0	22.4	5.3	All Genes
Post-transcriptional (2)	6.1	5.5	8.4	6.0	10.7	
Undetermined or ambiguous mechanism (3)	1.5	1.2	0.0	1.9	6.9	
No protein change (4)	88.4	90.6	40.0	68.2	73.0	
Transcriptional (1)	34.6	29.9	86.7	70.6	19.6	DE (iTRAQ) genes
Post-transcriptional (2)	52.5	58.0	13.3	23.5	54.9	
Undetermined or ambiguous mechanism (3)	12.9	12.1	0.0	5.9	25.5	
