# Supplementary material for: Prediction of Gene Activity in Early B Cell Development Based on an Integrative Multi-Omics Analysis
Source: J Proteomics Bioinform. Author manuscript; Available in PMC 2014 Dec 24. (PMC4276347; doi:10.4172/jpb.1000302)
Supplement: Supplemetary files [file NIHMS588429-supplement-Supplemetary_files.zip › Legends for Supplemental Figures and Tables.pdf]

Legends for Supplemental Figures and Tables

Supplemental Figure 1: Comparison of $cRPKM_P$- and MACS-based active chromatin prediction methods

Two representative examples where MACS-based promoter prediction failed to categorize promoter regions as active, despite having a significant enrichment of active chromatin modifications. Each panel shows the H3ac (red) and H3K4me3 (blue) abundance, and above that promoters predicted to be active by the $cRPKM_P$ (> 8) method and by the MACS method are indicated by black bars for each cell line. Directional tick marks on the gene depictions indicate direction of transcription. The tall shaded boxes indicate the promoter regions of each gene.

Supplemental Figure 2: Validation of $cRPKM_P$ prediction method

Graph showing the relative frequency distribution of cRPKM values (A) and box and whiskers plot representing the quartiles of cRPKM values (B) for all protein-coding RefSeq promoters (+/- 200 bp of the TSS), regions 2 kb upstream of RefSeq protein-coding promoters, and 1,000,000 random genomic intervals in pre-pro-B and pro-B cells. Frequency was assessed at increments of 0.1 (A), and regions with cRPKM = 0 are not represented in (A) but are included in (B). The Y-axis in (B) is

cRPKM on a $\log_{10}$ scale.

Supplemental Figure 3: Statistical validation of cRPKM method

Random genomic intervals with $cRPKM_R$ > 8 in pre-pro-B cells (A) and pro-B cells (B), and random genomic intervals with cRPKM < 8 in pre-pro-B cells (C) and pro-B cells (D) were analyzed for enrichment at RefSeq protein-coding TSS using the GenometriCorr R package [28]. The summary graphical output from this program is shown in panels (A-D). The black trend line indicates the frequency (Y-axis) with which a tested fragment (random intervals of $cRPKM_R$ > 8 or $cRPKM_R$ < 8) is found to be at a relative distance from the nearest RefSeq promoter element (0 indicates closest to the promoter and 0.5 the farthest possible). The trend line is generated by a large sliding window which averages frequencies over a large number of bins, thus this line only reaches '0' when large genomic distances have a zero value (as in A and B). Note the nearly 20-fold greater scale of the Y-axis in (A and B) versus (C and D). The heat map display of each graph indicates the range of correlation for that individual analysis by bins. The results of the statistical analyses are summarized to the right of the graphical summaries adjacent to panel (B) for $cRPKM_R$ > 8 and to the right of panel (D) for $cRPKM_R$ < 8. (E) The random genomic intervals in (A), (B), (C), and (D) were analyzed for enrichment at protein-coding RefSeq promoters using the Genomic Hyperbrowser [44]. The results from this analysis are shown.

Supplemental Figure 4: Relationship between $cRPKM_P$ and ongoing transcription (GRO-seq).

Plot of $cRPKM_P$ values of genes in the top quartile of RPKM (GRO-seq) values (red points) or the bottom quartile of RPKM values (blue points) in pre-pro-B cells (A) and pro-B cells (B). Points are plotted on the X-axis based on their rank within their respective quartiles, from lowest (left) to highest (right). The olive line indicates $cRPKM_P$ = 8.

Supplemental Figure 5: Relationship between MACS-based predicted promoter class and ongoing transcription (GRO-seq)

(A and B) Genes categorized based on their MACS-based predicted promoter class ('active' or 'inactive') are shown in box and whiskers plots representing the quartiles of GRO-seq values plotted versus GRO-seq transcription levels (RPKM on a log_{10} scale) in pre-pro-B cells (A) and pro-B cells (B). In these representations, all protein-coding genes in RefSeq are represented, including genes with RPKM = 0. (C and D) Graphs showing the relative frequency distribution of GRO-seq transcription levels [log_{10}(RPKM)] for each predicted promoter class (see color key) in pre-pro-B cells (C) and pro-B cells (D). (E and F) Graphs showing the relative frequency distribution of the differential ratio of GRO-seq transcription levels [$log_{10}(RPKM_{pro\text{-}B}/RPKM_{pre\text{-}pro\text{-}B})$] for each predicted chromatin class, with the magenta box of (E) magnified in (F). In these representations, frequency was assessed at increments of 0.1, and genes with RPKM = 0 are not included (C-F).

Supplemental Figure 6: Relationship between MACS predicted promoter class and steady state mRNA levels (RNA-seq)

(A) Genes categorized based on their MACS-based predicted promoter class ('active in both' or 'inactive in both') are shown in box and whiskers plots representing the quartiles of RNA-seq values plotted versus RNA-seq transcription levels (FPKM on a log_{10} scale) in pre-pro-B cells (A) and pro-B cells (B). In these representations, all protein-coding genes in RefSeq are considered, including genes with FPKM = 0. (C and D) Graphs showing the relative frequency distribution of RNA-seq transcription levels [log_{10}(FPKM)] for each predicted promoter class (see color key) in pre-pro-B cells (C) and pro-B cells (D). (E and F) Graphs showing the relative frequency distribution of the differential ratio of RNA-seq steady state mRNA levels [$log_{10}(FPKM_{pro\text{-}B}/FPKM_{pre\text{-}pro\text{-}B})$] for each predicted

chromatin class, with the magenta box of (E) magnified in (F). In these representations, frequency was assessed at increments of 0.1, and genes with RPKM = 0 are not included (C-F).

Supplemental Figure 7: GRO-seq and RNA-seq comparison

The ongoing transcription (RPKM) and steady state mRNA (FPKM) levels for the $cRPKM_P$-based predicted promoter classes: ‘active in both’ (A, B), ‘inactive in both’ (C, D), ‘exclusively pre-pro-B’ (E, F), ‘exclusively pro-B’ (G, H), and all genes (I, J) in pre-pro-B cells (A, C, E, G, I) and pro-B cells (B, D, F, H, J) are plotted as a contour plot (color scale indicates density of data points). Lines drawn at RPKM = -1 and FPKM = 0 were used to demarcate plot quadrants. The percent of genes in each quadrant was calculated and shown in each quadrant. Genes with ‘0’ values for either (or both) RPKM and FPKM were excluded from these plots.

Supplemental Figure 8: Differentially unstable genes

(A and B) Plots comparing ongoing transcription (RPKM) and steady state mRNA levels (FPKM) of genes in the ‘active in both’ predicted promoter class in pre-pro-B (A) and pro-B (B) cells (as in Supplemental Figure 7A and B). (C) Genes in the highlighted boxes in (A, orange) and (B, burgundy) are plotted by their differential expression at the ongoing transcriptional $[\log_{10}(RPKM_{pro\text{-}B}/RPKM_{pre\text{-}pro\text{-}B})]$ and steady state mRNA $[\log_{10}(FPKM_{pro\text{-}B}/FPKM_{pre\text{-}pro\text{-}B})]$ levels. Genes that are in both highlighted boxes of (A) and (B) are colored blue and genes that are only in one of the highlighted boxes, in either (A) or (B), are colored based on which box they are exclusively in.

Supplemental Figure 9: Relationship between MACS-based predicted promoter classes and FPKM/RPKM ratio

Graphs showing the relative frequency distribution of FPKM/RPKM ratios $[\log_{10}(FPKM/RPKM)]$ for

each MACS-based predicted promoter class (see color key) in pre-pro-B cells (A) and pro-B cells (B). Plot displaying the differential ratio of $\log_{10}(FPKM_{pro\text{-}B}/RPKM_{pro\text{-}B})/(FPKM_{pre\text{-}pro\text{-}B}/RPKM_{pre\text{-}pro\text{-}B})]$ for each MACS-based predicted promoter class (C). Frequency was assessed at increments of 0.1, and genes with zero value for either RPKM or FPKM were excluded from this analysis (A-C).

Supplemental Figure 10: Relationship between MACS-based predicted chromatin status and differential protein abundance

Histograms of differential protein abundance $[\log_2(iTRAQ_{pro\text{-}B/pre\text{-}pro\text{-}B})]$ for genes of the ‘active in both’ (A), ‘inactive in both’ (B), ‘exclusively pre-pro-B’ (C), and ‘exclusively pro-B’ (D) predicted promoter classes. The shaded region of the plot represents non-differentially abundant proteins and the unshaded region indicates differentially abundant proteins based on a 1.5 fold difference threshold. Each bar on the plots represents a bin with a range of 0.25.

Supplemental Figure 11: Magnitudes of differential steady-state mRNA (RNA-seq) and differential protein (iTRAQ) from post-transcriptional and transcriptional regulatory mechanisms

(A and B) Genes whose protein products have a differential levels (+/- 1.5-fold change in abundance) in pre-pro-B versus pro-B cells were grouped into four categories (‘transcriptional down’ regulated, ‘transcriptional up’ regulated, ‘post-transcriptional down’ regulated, and ‘post-transcriptional up’ regulated) based on whether the differential protein level appeared explained by post-transcriptional or transcriptional mechanisms $[\log_2(RPKM_{pro\text{-}B}/RPKM_{pre\text{-}pro\text{-}B})$ between -1 and 1, or <-1, or >1] and the direction of the differential expression (see Supplemental Table 1). Box and whiskers plot show the differential steady-state levels of their mRNAs $[\log_2(FPKM_{pro\text{-}B}/FPKM_{pre\text{-}pro\text{-}B})]$ (A) and proteins $[\log_2(iTRAQ_{pro\text{-}B/pre\text{-}pro\text{-}B})]$ (B), as quartiles. (C and D) The average fold change for the mRNA (C) and

protein (D) of the genes in the groups described in (A) and (B). Post-transcriptional regulation versus transcriptional regulation demonstrated statistically significant differences for both RNA and protein abundances (WMW $p < 0.001$).

Supplemental Figure 12: Relationship between steady differential state mRNA (RNA-seq) and differential protein (iTRAQ) levels

Differential steady state mRNA levels [$\log_2(FPKM_{pro\text{-}B}/FPKM_{pre\text{-}pro\text{-}B})$, X-axis] and differential steady state protein levels [$\log_2(iTRAQ_{pro\text{-}B/pre\text{-}pro\text{-}B})$, Y-axis] for all genes in which protein was detected by iTRAQ-based proteome quantification (irrespective of chromatin prediction group) are shown in a contour plot (color scale indicates density of data points). The arrow indicates 2410004B18Rik.

Supplemental Table 1: Genes in regulatory classes and groups

All genes for which protein products were identified by iTRAQ-based proteome quantification, and in each predicted promoter class, were classified in regulatory combinations based on their differential levels of ongoing transcription (GRO-seq), steady state mRNA (RNA-seq), and protein (iTRAQ-based proteome quantification). These 27 regulatory combinations were sorted into broader regulatory groupings. Fold change thresholds of differential ongoing transcriptional, steady state mRNA, and protein levels were set at 2, 2, and 1.5, respectively. Numbers and percentages of genes found to be in each group are presented for the predicted promoter classes and all genes.

Supplemental Table 2: Established and novel differentially abundant genes in this developmental model

Examples of genes that were previously established to be differentially expressed in our early B lymphopoiesis model (upper six lines) and genes shown newly in this study to be differentially

expressed (lower four lines) .The differential expression status of transcription, steady state mRNA, and protein levels are indicated along with the proposed mode of regulation. Actual values can be found in Supplemental Data File 3.

Supplemental Data File information

Supplemental Data File 1: Genome wide analysis parameters

This spreadsheet contains the parameters used for data analysis of ChIP-seq, GRO-seq, and RNA-seq data.

Supplemental Data File 2: Massively parallel sequencing library metrics

This spreadsheet contains the sequencing library size and number of mapped reads for ChIP-seq, GRO-seq, and RNA-seq data.

Supplemental Data File 3: Integrated multi-omics data

This spreadsheet contains the cRPKM, RPKM, FPKM, FPKM/RPKM, and iTRAQ data for all protein-coding genes in RefSeq and their respective predicted promoter classifications.

Data access

Data used in this work can be found on Galaxy and GEO. All RNA-seq data, including fastq reads, aligned reads, and quantitative abundance measurements can be found on Galaxy. Bedgraph files corresponding to all ChIP-seq, GRO-seq, and RNA-seq data are available on Galaxy as well.

https://usegalaxy.org/u/thereddylab/p/prediction-of-gene-activity-based-on-an-integrative-multi-omics-analysis

Raw fastq RNA-seq reads and transcript quantification data are hosted on GEO under the accession: GSE52450.

http://www.ncbi.nlm.nih.gov/geo/query/acc.cgi?acc=GSE52450

Acknowledgements

We are thankful to members of the Reddy laboratory for helpful discussions and on-going dialogue. We thank Tai-Chung Huang, M.D.-Ph.D. for proteome sample preparation assistance and helpful discussions. We are grateful to the Galaxy Community for developing and maintaining Cloudman as well as the numerous tools employed in our analyses. We are especially thankful to Dave Clements of the Galaxy Project, who initially introduced us to performing NGS analysis with Galaxy Cloudman.
