# Supplementary material for: Prediction of Gene Activity in Early B Cell Development Based on an Integrative Multi-Omics Analysis
Source: J Proteomics Bioinform. Author manuscript; Available in PMC 2014 Dec 24. (PMC4276347; doi:10.4172/jpb.1000302)
Supplement: Supplemetary files [file NIHMS588429-supplement-Supplemetary_files.zip › Supplemental Figures and Tables.pdf]

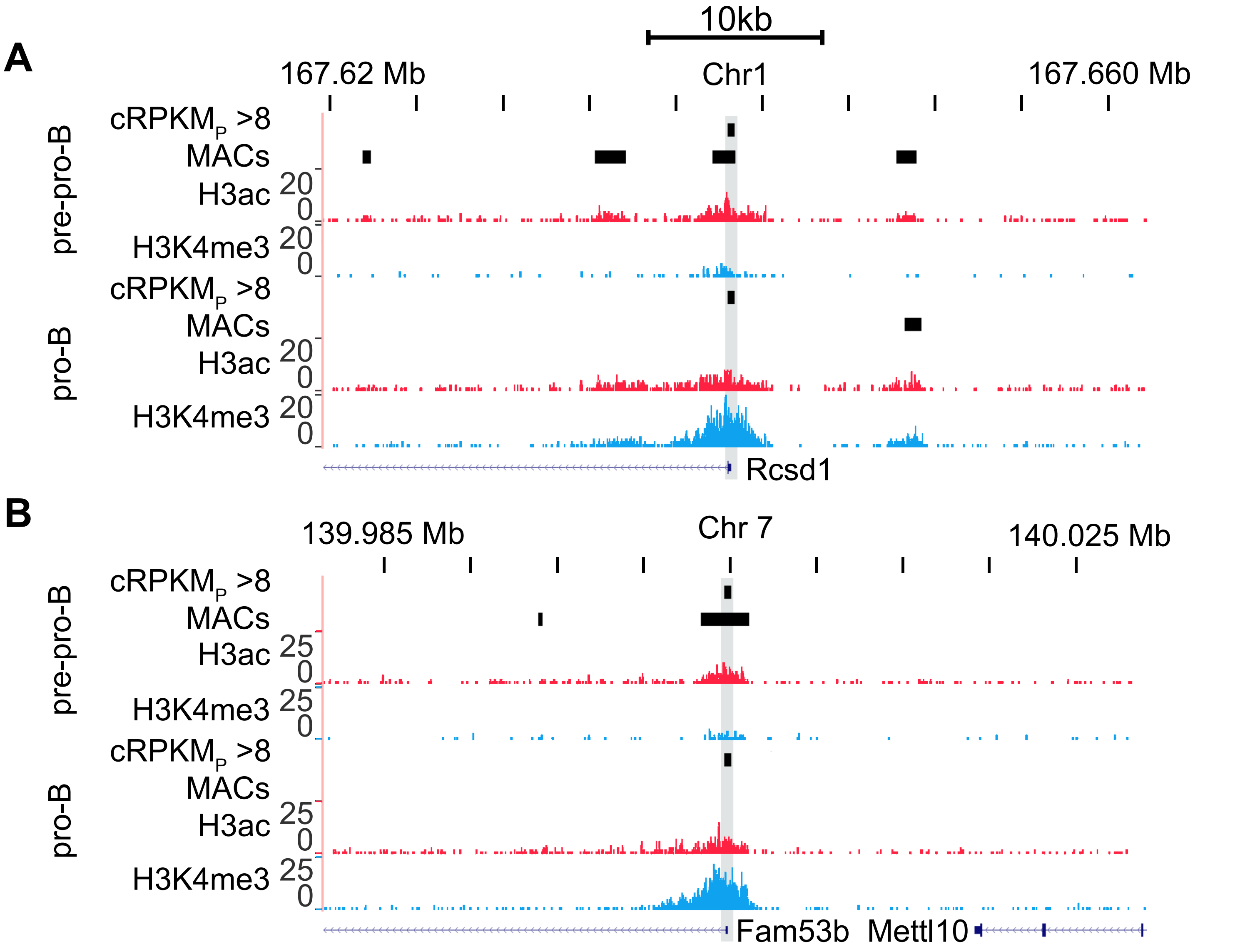
A
10kb
167.62 Mb
Chr1
167.660 Mb
pre-pro-B
cRPKM$_P$ >8
MACs
H3ac
20
0
H3K4me3
20
0
pro-B
cRPKM$_P$ >8
MACs
H3ac
20
0
H3K4me3
20
0
Rcsd1
B
139.985 Mb
Chr 7
140.025 Mb
pre-pro-B
cRPKM$_P$ >8
MACs
H3ac
25
0
H3K4me3
25
0
pro-B
cRPKM$_P$ >8
MACs
H3ac
25
0
H3K4me3
25
0
Fam53b
Mettl10

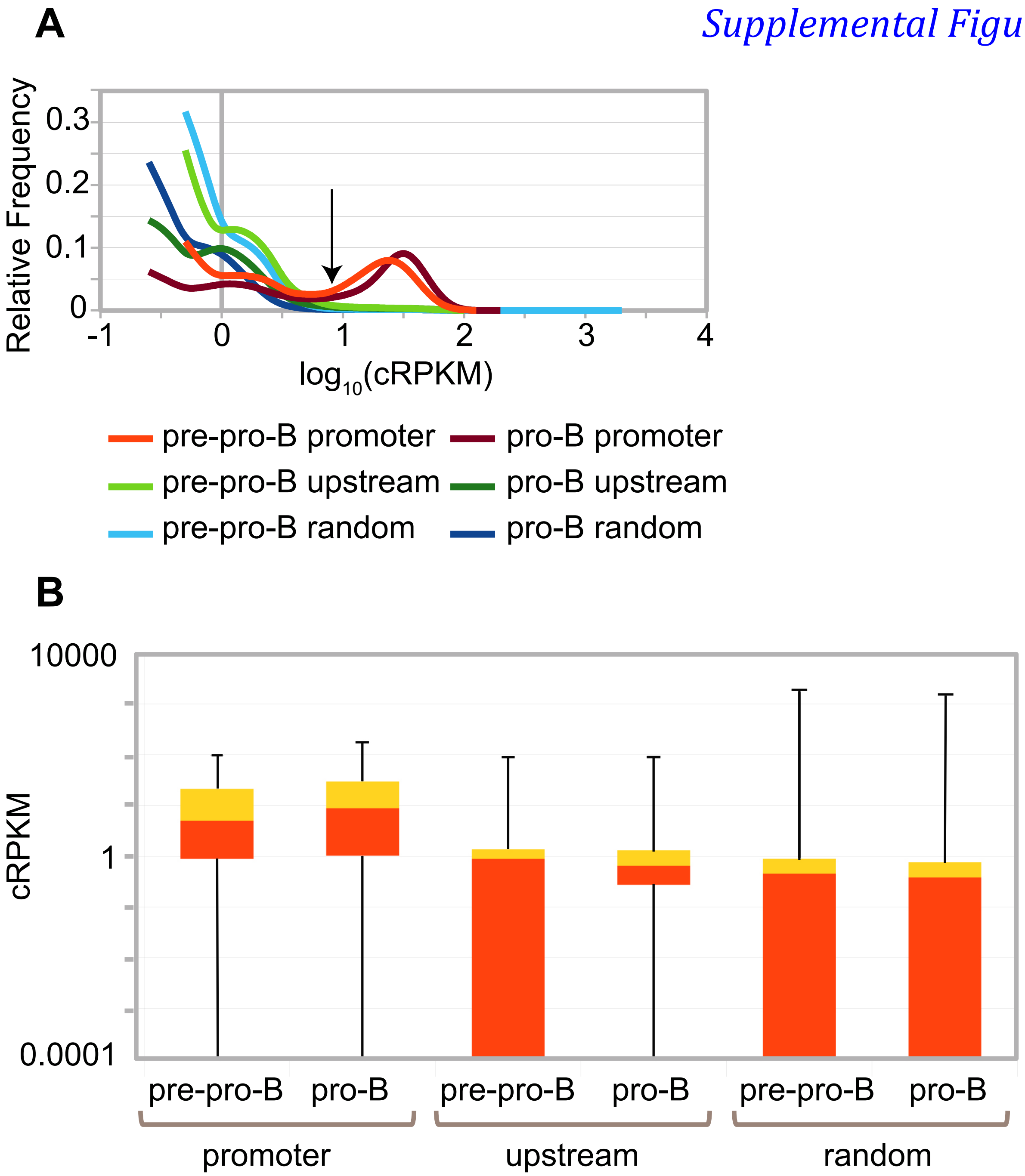
A
Relative Frequency
0.3
0.2
0.1
0
-1
0
1
2
3
4
log10(cRPKM)
pre-pro-B promoter
pro-B promoter
pre-pro-B upstream
pro-B upstream
pre-pro-B random
pro-B random
B
10000
1
0.0001
cRPKM
pre-pro-B
pro-B
pre-pro-B
pro-B
pre-pro-B
pro-B
promoter
upstream
random

Supplemental Figure 3: Heydarian et al.

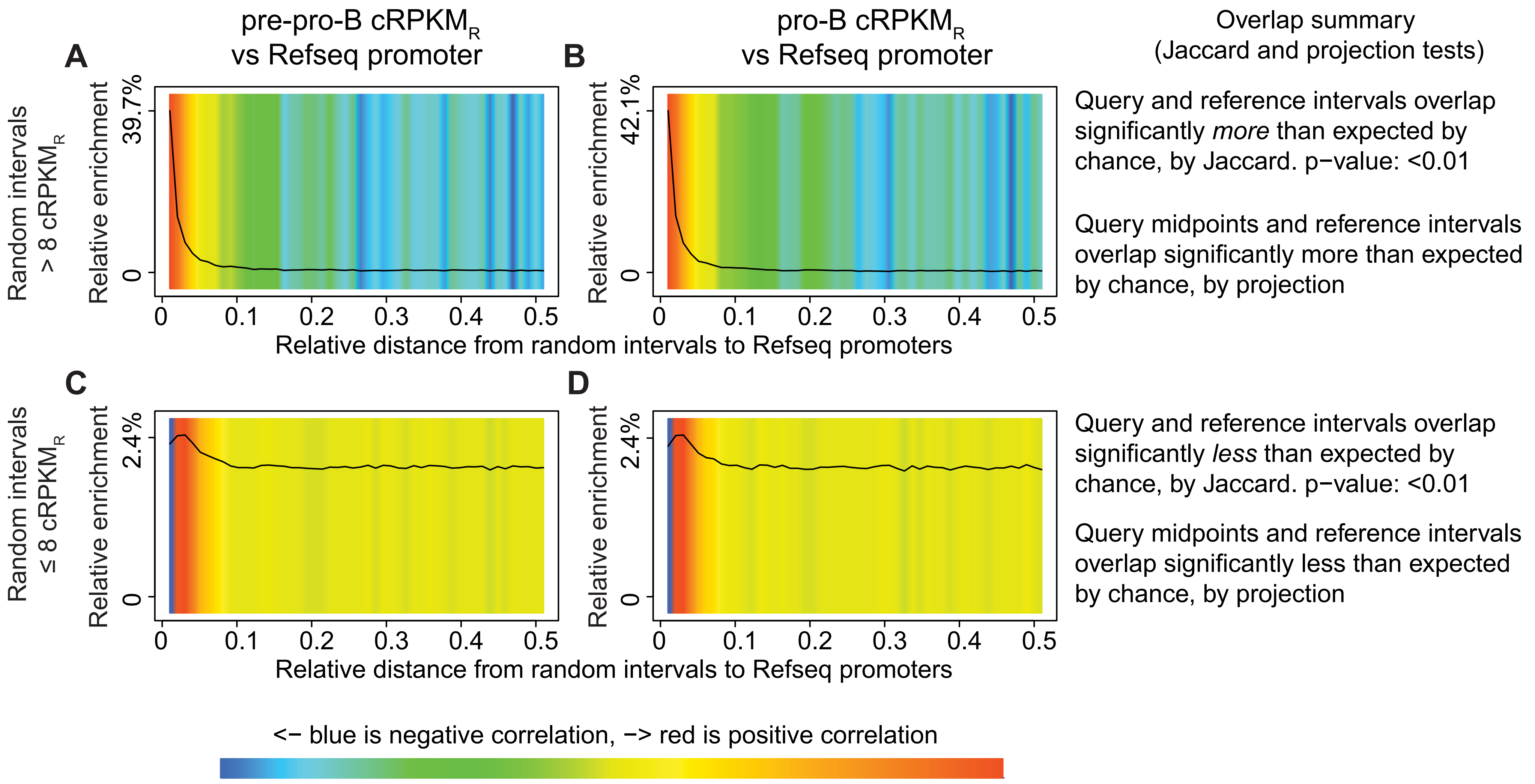

E

	Overlap with Refseq promoters -- Genomic Hyperbrowser answer	Genomic Hyperbrowser p-value
Random intervals in pre-pro-B, cRPKM$_R$ >8	Yes -- the data suggest this.	0.004975
Random intervals in pro-B, cRPKM$_R$ >8	Yes -- the data suggest this.	0.004975
Random intervals in pre-pro-B, cRPKM$_R$ <8	No support from data for this conclusion.	1.0
Random intervals in pro-B, cRPKM$_R$ <8	No support from data for this conclusion.	1.0

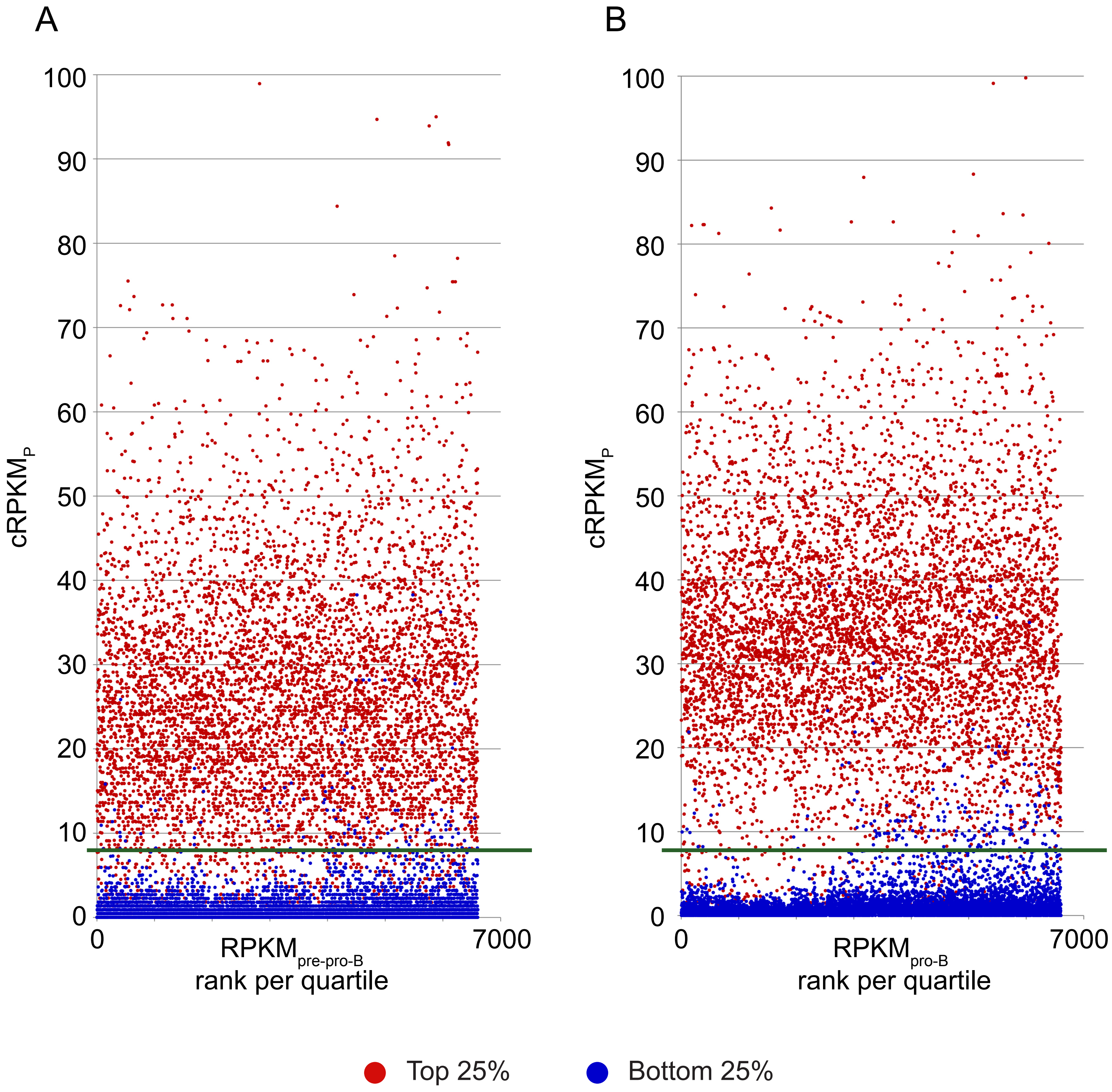
A
B
100
90
80
70
60
50
40
30
20
10
0
cRPKM$_P$
0
7000
RPKM$_{pre-pro-B}$
rank per quartile
RPKM$_{pro-B}$
rank per quartile
Top 25%
Bottom 25%

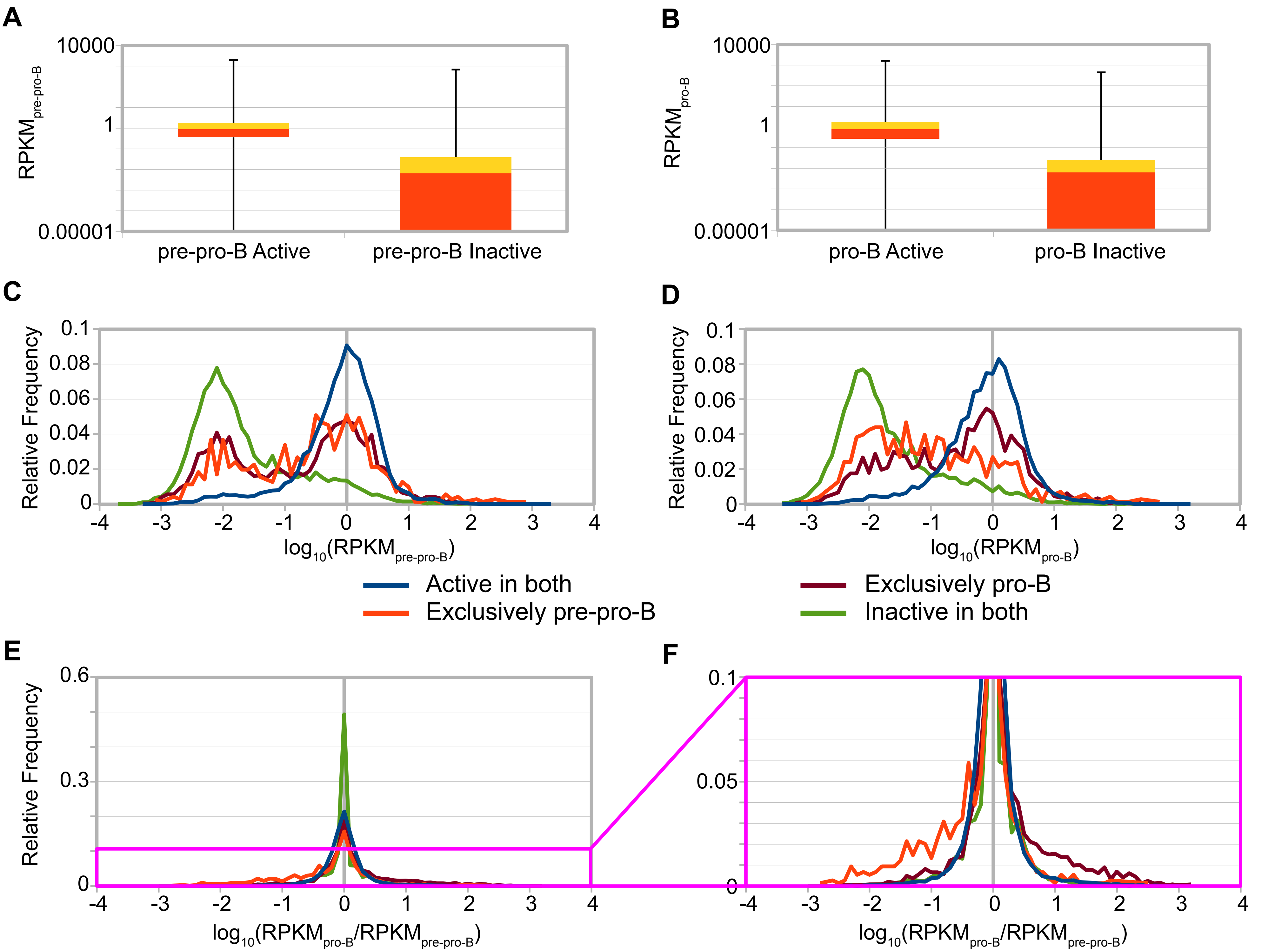
A
10000
1
0.00001
RPKM pre-pro-B
pre-pro-B Active
pre-pro-B Inactive
B
10000
1
0.00001
RPKM pro-B
pro-B Active
pro-B Inactive
C
0.1
0.08
0.06
0.04
0.02
0
Relative Frequency
-4 -3 -2 -1 0 1 2 3 4
log10(RPKM pre-pro-B)
D
0.1
0.08
0.06
0.04
0.02
0
Relative Frequency
-4 -3 -2 -1 0 1 2 3 4
log10(RPKM pro-B)
Active in both
Exclusively pre-pro-B
Exclusively pro-B
Inactive in both
E
0.6
0.3
0
Relative Frequency
-4 -3 -2 -1 0 1 2 3 4
log10(RPKM pro-B/RPKM pre-pro-B)
F
0.1
0.05
0
-4 -3 -2 -1 0 1 2 3 4
log10(RPKM pro-B/RPKM pre-pro-B)

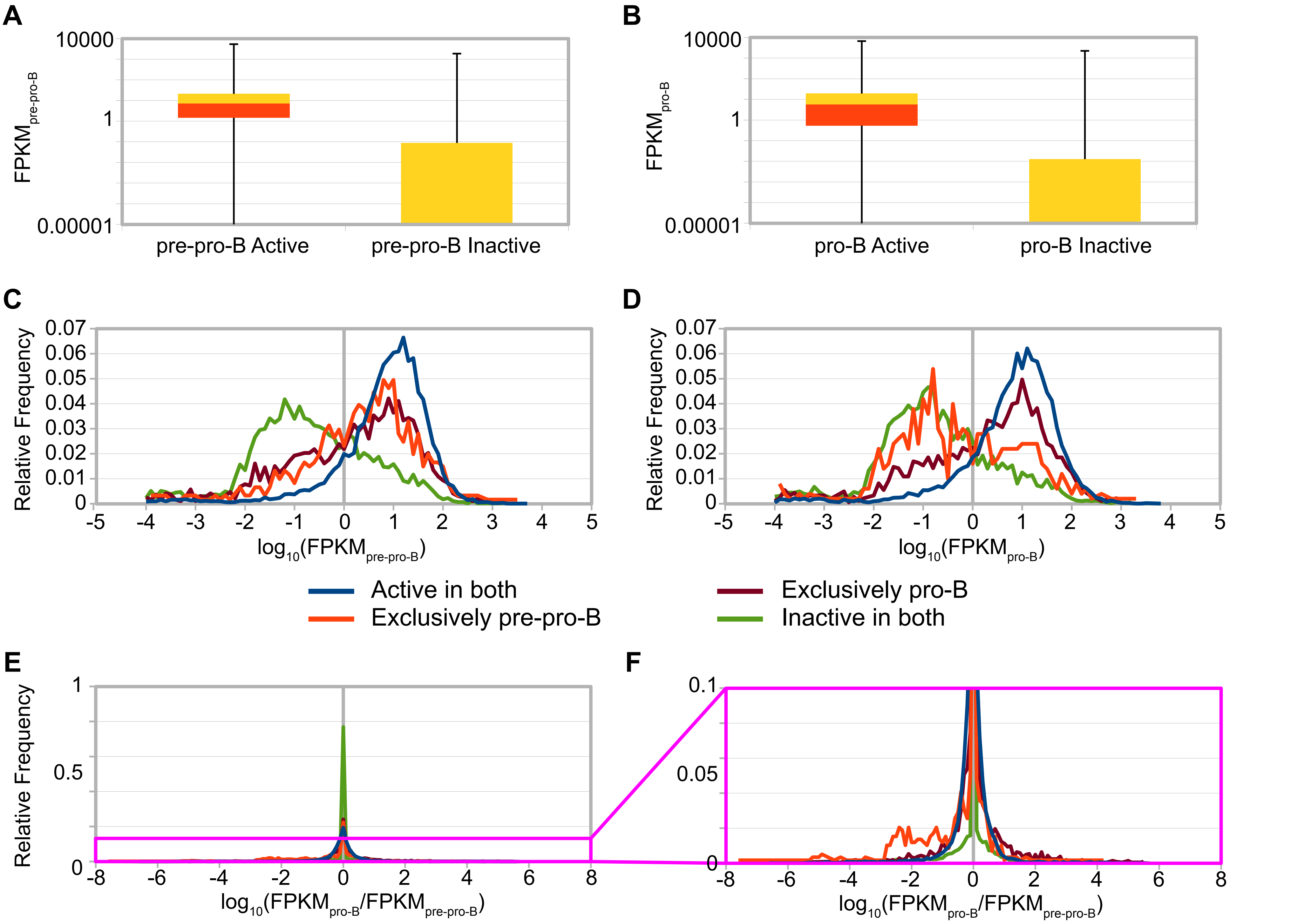
A
10000
1
0.00001
FPKMpre-pro-B
pre-pro-B Active
pre-pro-B Inactive
B
10000
1
0.00001
FPKMpro-B
pro-B Active
pro-B Inactive
C
Relative Frequency
0.07
0.06
0.05
0.04
0.03
0.02
0.01
0
-5 -4 -3 -2 -1 0 1 2 3 4 5
log10(FPKMpre-pro-B)
D
Relative Frequency
0.07
0.06
0.05
0.04
0.03
0.02
0.01
0
-5 -4 -3 -2 -1 0 1 2 3 4 5
log10(FPKMpro-B)
Active in both
Exclusively pre-pro-B
Exclusively pro-B
Inactive in both
E
Relative Frequency
1
0.5
0
-8 -6 -4 -2 0 2 4 6 8
log10(FPKMpro-B/FPKMpre-pro-B)
F
0.1
0.05
0
-8 -6 -4 -2 0 2 4 6 8
log10(FPKMpro-B/FPKMpre-pro-B)

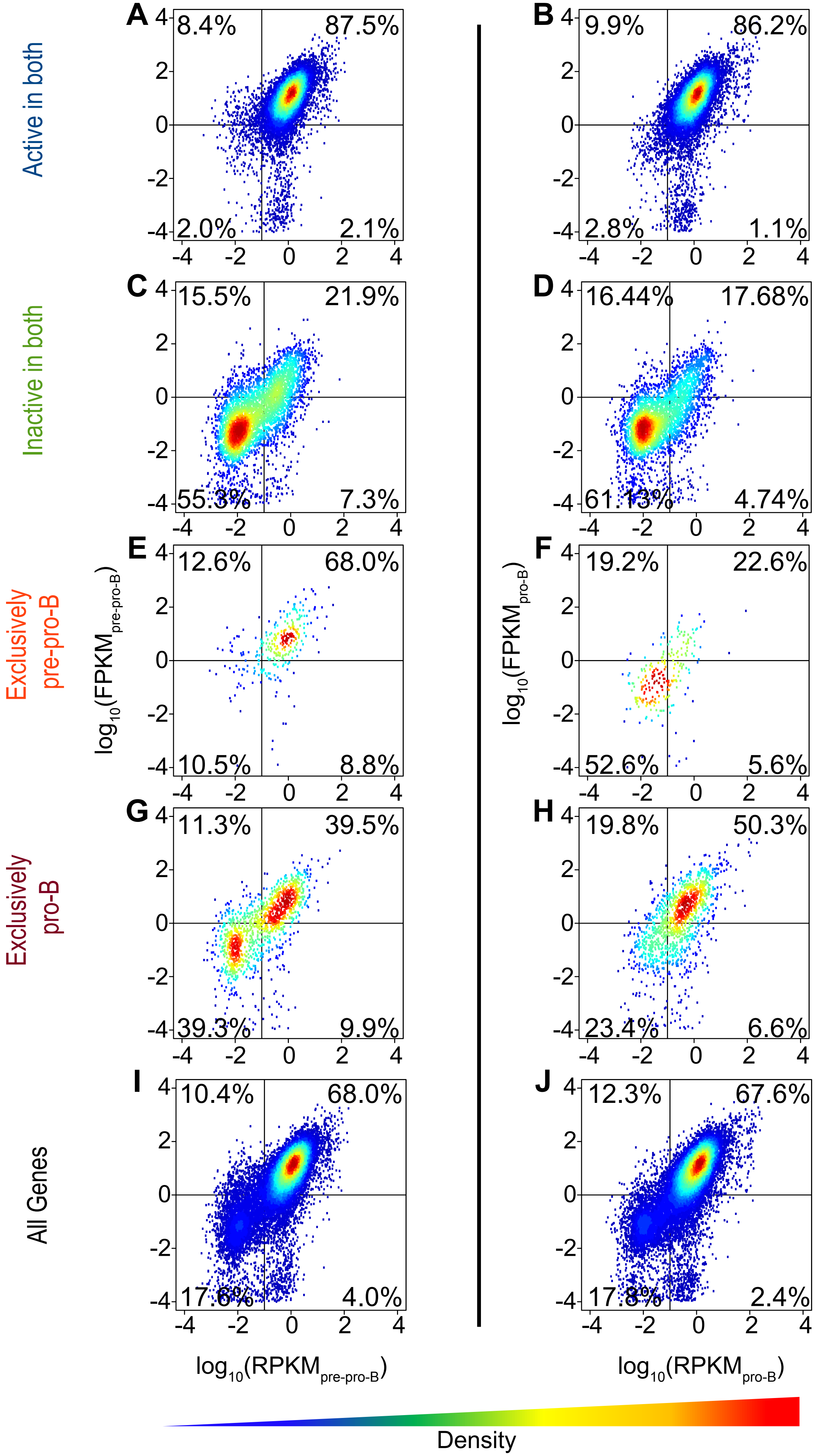

Active in both
Inactive in both
Exclusively pre-pro-B
Exclusively pro-B
All Genes
A
8.4%
87.5%
2.0%
2.1%
B
9.9%
86.2%
2.8%
1.1%
C
15.5%
21.9%
55.3%
7.3%
D
16.44%
17.68%
61.13%
4.74%
E
12.6%
68.0%
10.5%
8.8%
F
19.2%
22.6%
52.6%
5.6%
G
11.3%
39.5%
39.3%
9.9%
H
19.8%
50.3%
23.4%
6.6%
I
10.4%
68.0%
17.6%
4.0%
J
12.3%
67.6%
17.8%
2.4%
log10(FPKMpre-pro-B)
log10(FPKMpro-B)
log10(RPKMpre-pro-B)
log10(RPKMpro-B)
Density

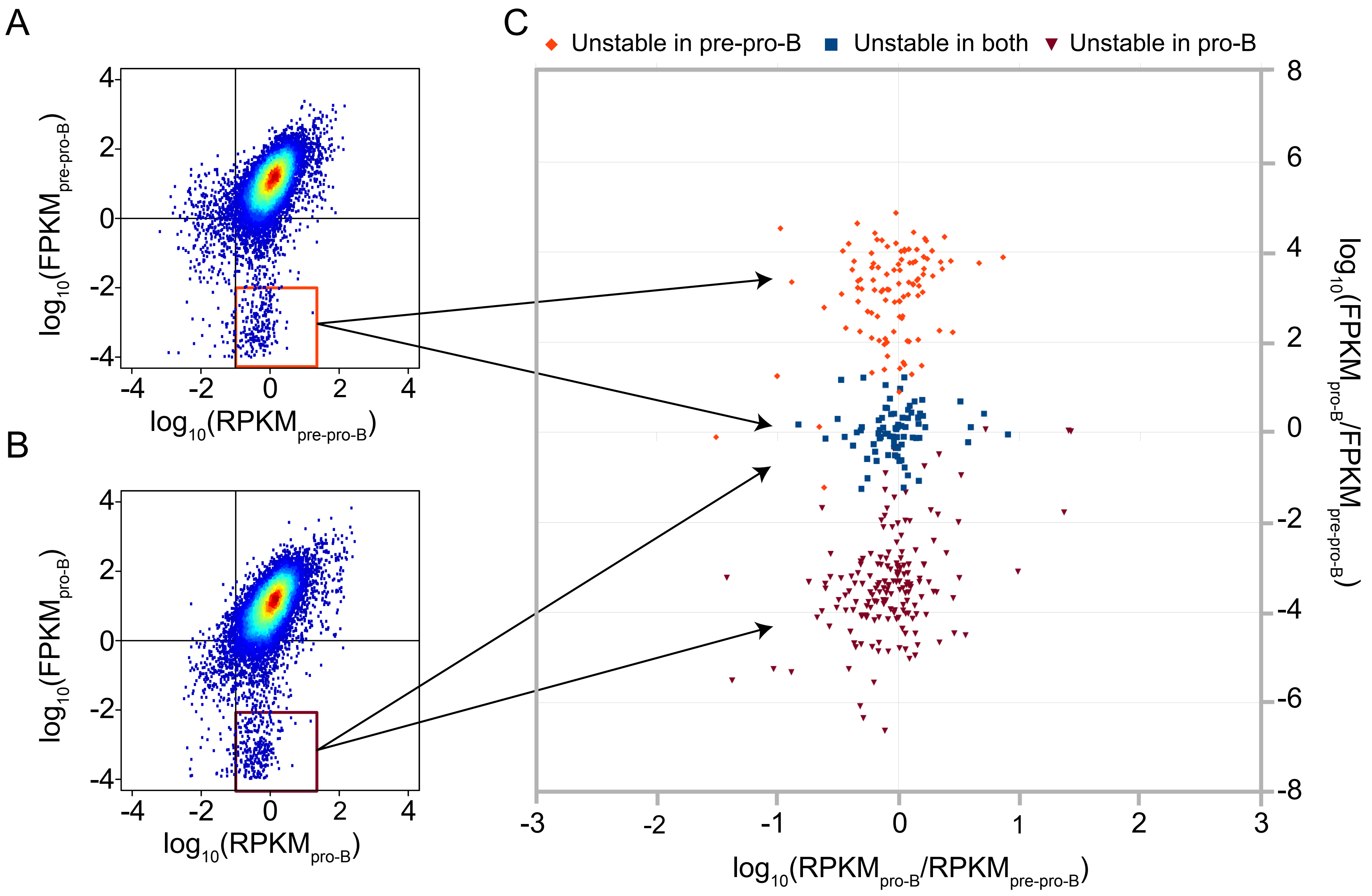
A
B
C
Unstable in pre-pro-B
Unstable in both
Unstable in pro-B
log10(FPKMpre-pro-B)
log10(RPKMpre-pro-B)
log10(FPKMpro-B)
log10(RPKMpro-B)
log10(FPKMpro-B/FPKMpre-pro-B)
log10(RPKMpro-B/RPKMpre-pro-B)

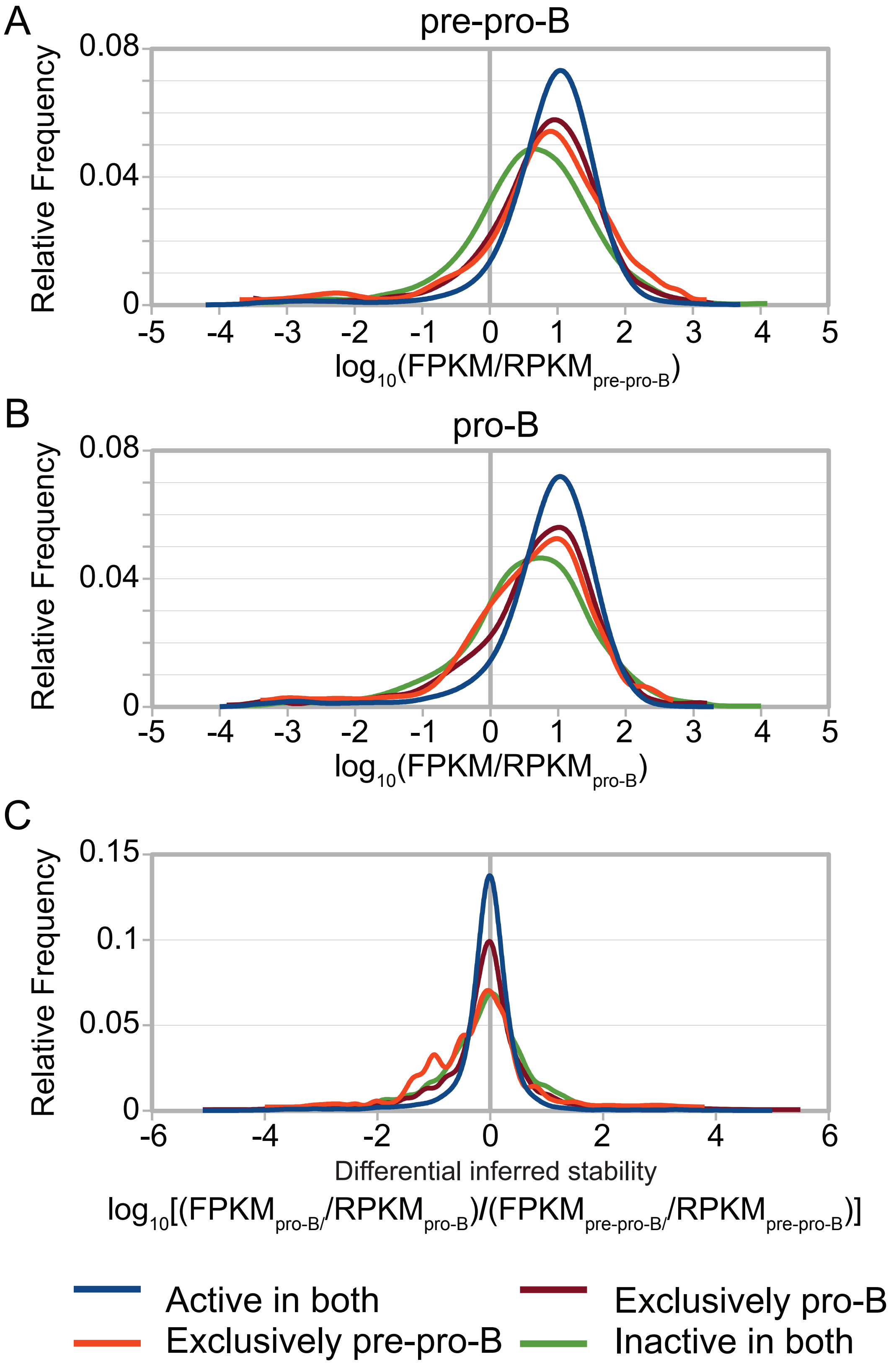
A
pre-pro-B
Relative Frequency
0.08
0.04
0
-5 -4 -3 -2 -1 0 1 2 3 4 5
log10(FPKM/RPKMpre-pro-B)
B
pro-B
Relative Frequency
0.08
0.04
0
-5 -4 -3 -2 -1 0 1 2 3 4 5
log10(FPKM/RPKMpro-B)
C
Relative Frequency
0.15
0.1
0.05
0
-6 -4 -2 0 2 4 6
Differential inferred stability
log10[(FPKMpro-B/RPKMpro-B)/(FPKMpre-pro-B/RPKMpre-pro-B)]
Active in both
Exclusively pro-B
Exclusively pre-pro-B
Inactive in both

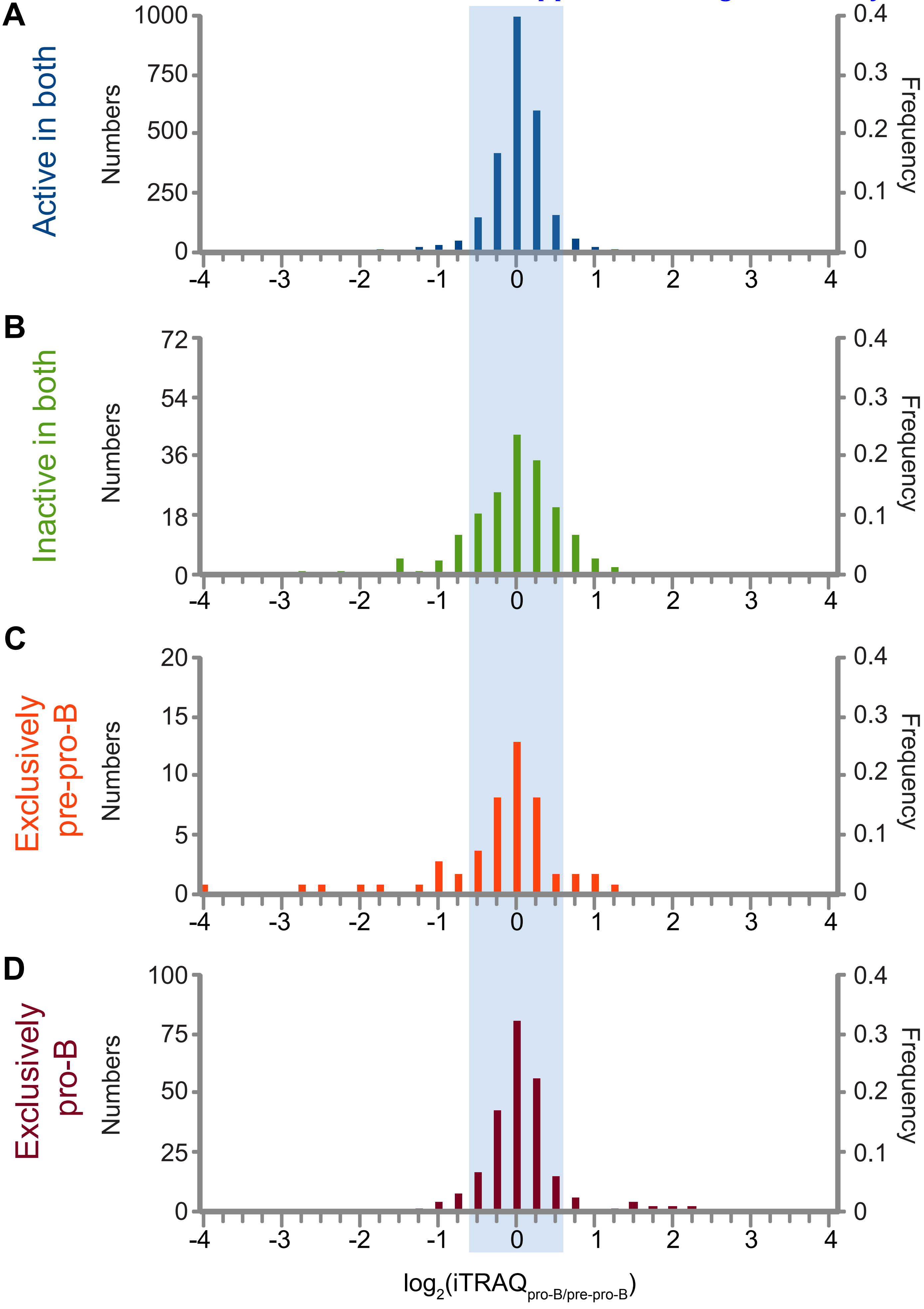
A
Active in both
Numbers
1000
750
500
250
0
0.4
0.3
0.2
0.1
0
Frequency
-4
-3
-2
-1
0
1
2
3
4
B
Inactive in both
72
54
36
18
C
Exclusively pre-pro-B
20
15
10
5
D
Exclusively pro-B
100
75
50
25
log2(iTRAQpro-B/pre-pro-B)

A

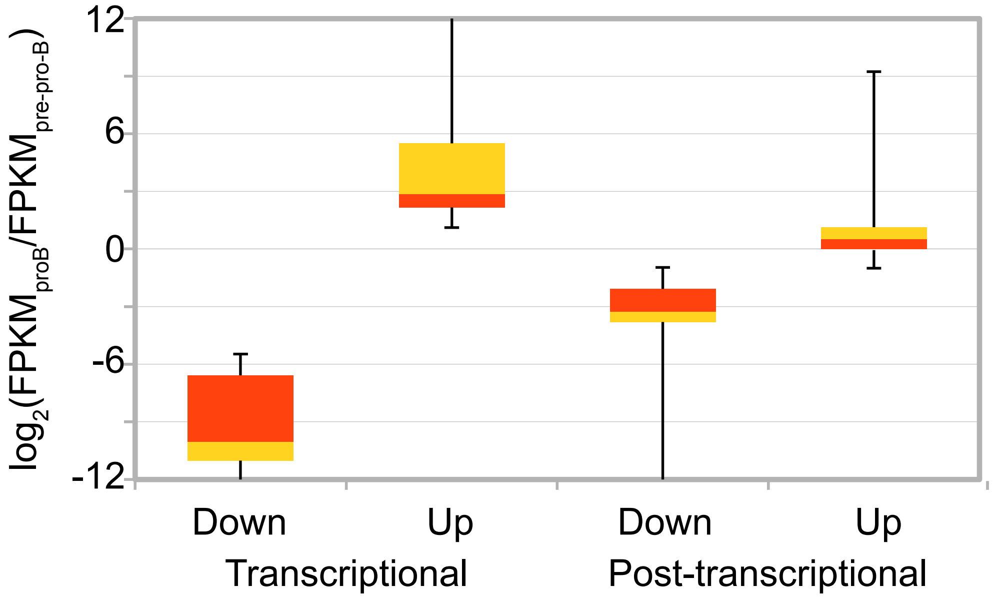

B

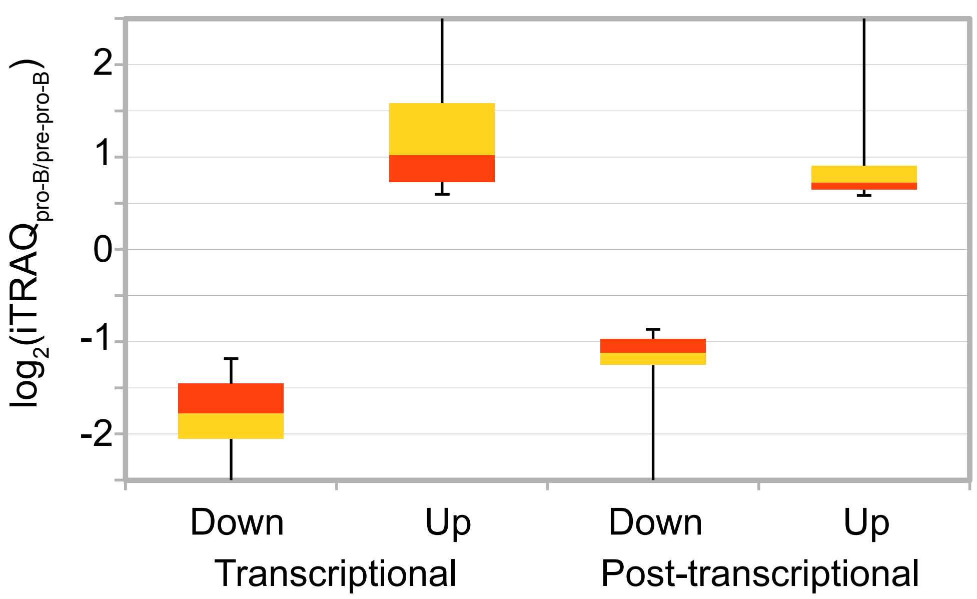

C

	mean $log_2(FPKM_{proB}/FPKM_{pre-pro-B})$
Transcriptional down	-4.77
Transcriptional up	4.00
Post-transcriptional down	-2.10
Post-transcriptional up	2.78

D

	mean $log_2(iTRAQ_{pro-B/pre-pro-B})$
Transcriptional down	-1.32
Transcriptional up	1.23
Post-transcriptional down	-0.95
Post-transcriptional up	0.84

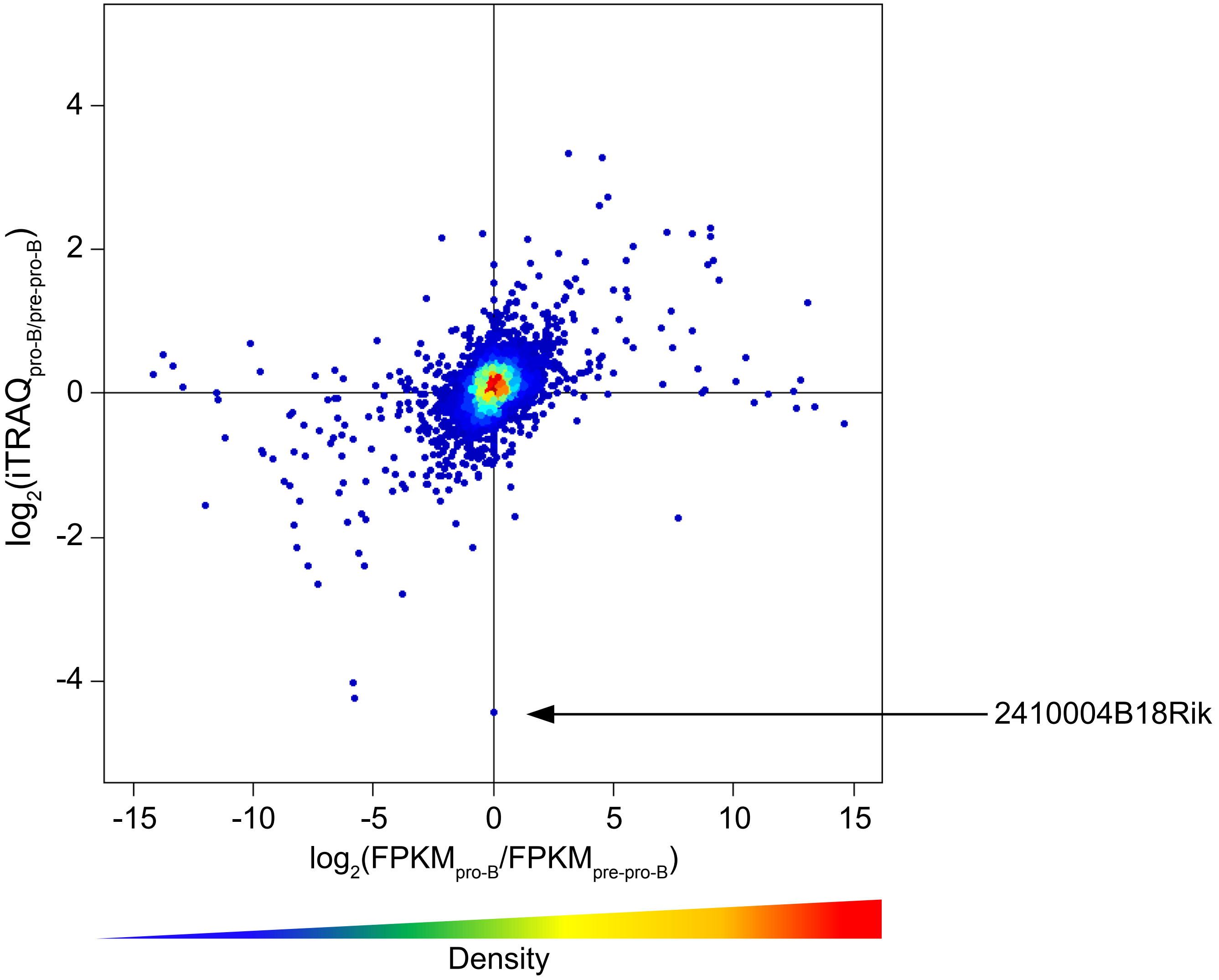
4
2
0
-2
-4
log2(iTRAQpro-B/pre-pro-B)
-15
-10
-5
0
5
10
15
log2(FPKMpro-B/FPKMpre-pro-B)
2410004B18Rik
Density

Supplemental Table 1: Heydarian et al.

	Regulatory combination			Number of genes					Percentage of class				
**Broad regulatory group**	**GRO-seq**	**RNA-seq**	**iTRAQ**	**All genes**	**Active in both**	**Exclusively pre-pro-B**	**Exclusively pro-B**	**Inactive in both**	**All genes**	**Active in both**	**Exclusively pre-pro-B**	**Exclusively pro-B**	**Inactive in both**
**Transcriptional (1)**	UP	UP	UP	68	41	0	24	3	2.17	1.46	0	22.43	1.59
	DOWN	DOWN	DOWN	58	38	13	0	7	1.85	1.35	52.00	0	3.70
**Post-transcriptional (2)**	no change	UP	UP	29	22	0	3	4	0.93	0.78	0	2.80	2.12
	no change	DOWN	DOWN	37	28	1	2	6	1.18	1.00	4.00	1.87	3.17
	no change	no change	UP	81	66	1	2	12	2.59	2.35	4.00	1.87	6.35
	no change	no change	DOWN	44	37	0	1	6	1.41	1.32	0	0.93	3.17
**Undetermined or ambiguous mechanism (3)**	UP	no change	UP	13	9	0	0	4	0.42	0.32	0	0	2.12
	DOWN	no change	DOWN	6	4	0	0	2	0.19	0.14	0	0	1.06
	UP	UP	DOWN	0	0	0	0	0	0	0	0	0	0
	UP	no change	DOWN	1	1	0	0	0	0.03	0.04	0	0	0
	UP	DOWN	UP	3	2	0	0	1	0.10	0.07	0	0	0.53
	UP	DOWN	DOWN	8	3	0	2	3	0.26	0.11	0	1.87	1.59
	no change	UP	DOWN	1	1	0	0	0	0.03	0.04	0	0	0
	no change	DOWN	UP	7	5	0	0	2	0.22	0.18	0	0	1.06
	DOWN	UP	UP	2	2	0	0	0	0.06	0.07	0	0	0
	DOWN	UP	DOWN	1	1	0	0	0	0.03	0.04	0	0	0
	DOWN	no change	UP	4	3	0	0	1	0.13	0.11	0	0	0.53
	DOWN	DOWN	UP	1	1	0	0	0	0.03	0.04	0	0	0
**No protein change (4)**	UP	UP	no change	74	63	0	6	5	2.36	2.24	0	5.61	2.65
	UP	no change	no change	91	76	0	6	9	2.91	2.71	0	5.61	4.76
	UP	DOWN	no change	9	7	0	2	0	0.29	0.25	0	1.87	0
	no change	UP	no change	214	195	0	3	16	6.84	6.94	0	2.80	8.47
	no change	no change	no change	2008	1896	1	38	73	64.17	67.52	4.00	35.51	38.62
	no change	DOWN	no change	220	187	1	8	24	7.03	6.66	4.00	7.48	12.7
	DOWN	UP	no change	7	6	0	1	0	0.22	0.21	0	0.93	0
	DOWN	no change	no change	66	58	1	6	1	2.11	2.07	4.00	5.61	0.53
	DOWN	DOWN	no change	76	56	7	3	10	2.43	1.99	28.00	2.80	5.29

Gene	Chromatin class	Differential expression (pro-B/pre-pro-B)			Regulatory mechanism
		GRO-seq	RNA-seq	iTRAQ	
Cd34	Exclusively pre-pro-B	down	down	down	Transcriptional
Flt3	Exclusively pre-pro-B	down	down	down	Transcriptional
Ikzf3	Exclusively pro-B	up	up	up	Transcriptional
Pou2af1	Exclusively pro-B	up	up	up	Transcriptional
Irf4	Exclusively pro-B	up	up	up	Transcriptional
Cst7	Exclusively pre-pro-B	down	down	down	Transcriptional
Arid3a	Active in both	down	down	down	Transcriptional
AB124611	Active in both	no change	down	down	Post-transcriptional – stability
Galnt11	Active in both	no change	down	up	Post-transcriptional – translational/protein turnover
2410004B18Rik	Active in both	no change	no change	down	Post-transcriptional – translational/protein turnover
